# Planktonic functional diversity changes in synchrony with lake ecosystem state

**DOI:** 10.1111/gcb.16485

**Published:** 2022-11-12

**Authors:** Duncan A. O'Brien, Gideon Gal, Stephen J. Thackeray, Shin‐ichiro S. Matsuzaki, Christopher F. Clements

**Affiliations:** ^1^ School of Biological Sciences University of Bristol Bristol UK; ^2^ Kinneret Limnological Laboratory Israel Oceanographic and Limnological Research Migdal Israel; ^3^ Lake Ecosystems Group UK Centre for Ecology and Hydrology Lancaster UK; ^4^ Biodiversity Division National Institute for Environmental Studies Tsukuba Japan

**Keywords:** aquatic, biodiversity, ecosystem functioning, indicator, management, trait‐based approach

## Abstract

Managing ecosystems to effectively preserve function and services requires reliable tools that can infer changes in the stability and dynamics of a system. Conceptually, functional diversity (FD) appears as a sensitive and viable monitoring metric stemming from suggestions that FD is a universally important measure of biodiversity and has a mechanistic influence on ecological processes. It is however unclear whether changes in FD consistently occur prior to state responses or vice versa, with no current work on the temporal relationship between FD and state to support a transition towards trait‐based indicators. There is consequently a knowledge gap regarding when functioning changes relative to biodiversity change and where FD change falls in that sequence. We therefore examine the lagged relationship between planktonic FD and abundance‐based metrics of system state (e.g. biomass) across five highly monitored lake communities using both correlation and cutting edge non‐linear empirical dynamic modelling approaches. Overall, phytoplankton and zooplankton FD display synchrony with lake state but each lake is idiosyncratic in the strength of relationship. It is therefore unlikely that changes in plankton FD are identifiable before changes in more easily collected abundance metrics. These results highlight the power of empirical dynamic modelling in disentangling time lagged relationships in complex multivariate ecosystems, but suggest that FD cannot be generically viable as an early indicator. Individual lakes therefore require consideration of their specific context and any interpretation of FD across systems requires caution. However, FD still retains value as an alternative state measure or a trait representation of biodiversity when considered at the system level.

## Graphical abstract

Using species‐level plankton trait and abundance data from five highly monitored lakes, we determined the timing of functional diversity change relative to ecosystem state/functioning. Simple cross‐correlation and novel convergent cross mapping techniques revealed that planktonic functional diversity was weakly associated with state (with individual lakes displaying unique relationships), but that both measures changed simultaneously. This implies functional diversity is not a leading indicator of state change but contains additional information absent in the state measures used here.

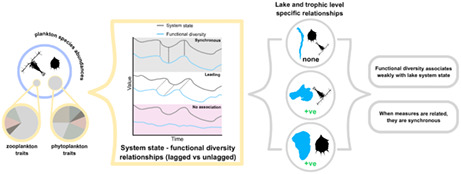

## 1 INTRODUCTION

Predicting oncoming ecosystem change is a vital first step in the management of both ecosystems and their associated resources. Ecosystem state or functioning is considered the critical target in this regard as its disruption can result in the loss of many ecosystem services upon which human societies are dependent (Rockström et al., 2009). Since the turn of the millennium, biodiversity has appeared as the principal determinant and indicator of both ecosystem state (Tilman et al., 2014) and resilience (Oliver et al., 2015) across a range of biomes and scales. Biodiversity itself is inherently multidimensional, consisting of taxonomic (i.e. species), functional (i.e. trait) and genetic components among others (Lyashevska & Farnsworth, 2012; Naeem et al., 2016). Despite this multidimensionality, focus has primarily been on the impact of the taxonomic component with consistent positive associations between species diversity and ecosystem functioning identified in multiple taxa and environments (Cardinale et al., 2006; Duffy et al., 2017). However, there is significant evidence that other biodiversity dimensions are as impactful on an ecosystem's function as species richness, particularly the diversity in traits (Cadotte et al., 2011; Mouillot et al., 2011).

Trait diversity as a predictor of function stems from the view that, ecologically, a species (or a community) is a collection of phenotypic traits that determines their temporal and spatial impacts on their surroundings and each other (McGill et al., 2006). Higher trait diversity consequently allows species to coexist and exploit a wider range of ecological niches (Fukami et al., 2005) that, in turn, increases the number of ecological functions/services performed by the community. Multiple studies support this view and present similarly strong associations between functional diversity and functioning measures (de Bello et al., 2010; Mouillot et al., 2011) with some further suggesting that functional diversity sufficiently outperforms species diversity measures as a predictor of ecosystem state change (Abonyi, Horváth, et al., 2018; Gagic et al., 2015). Functional diversity is thus increasingly being considered as an emergent property of complex systems (Bullock et al., 2022) regardless of the environmental conditions driving that diversity change. This emerging evidence in favour of functional diversity suggests that trait change can feasibly occur prior to changes in system state, and may represent a viable early warning of change without necessarily requiring the identification of driving variables.

Timing is central to managing ecosystems (Hastings, 2016); the optimal moment for ecological intervention varies depending on both disturbance severity and the specific system (Walker et al., 2014). Consequently, any monitoring strategy should include measures that provide sufficient ‘warning’ to enable appropriate planning and action. The pre‐emptive or anticipatory nature of an indicator is therefore key for managers when selecting from a suite of potential indicators (Dale & Beyeler, 2001) with functional diversity potentially fulfilling this consideration. Indeed, inclusion of trait information improves the robustness of ecological model predictions (Regos et al., 2019; Williams et al., 2021) and other early warning techniques (Clements & Ozgul, 2016). However, despite these suggestions and repeated claims that functional diversity changes following dramatic state changes (e.g. land use change—Edwards et al., 2014; lake regime shifts—Moi et al., 2021), there remains a need to confirm that diversity changes also occur prior to ecosystem state change, as required by indicator selection frameworks (Dale & Beyeler, 2001).

To date, only one example exists of the lagged association between functional and species diversity (Baker et al., 2021), although this work is solely a qualitative description of trends without any quantitative association and does not consider state/functioning. All other functional diversity research similarly neglects temporal relationships between measures. However, species diversity is not necessarily a reliable measure of state/functioning (Cardinale et al., 2000). It is likely functional diversity is also susceptible to the same caveats despite suggestions that the functional diversity–state relationship is shared across systems and scales (Cadotte et al., 2011; Gagic et al., 2015). It is similarly unclear where on the driver‐to‐state‐change sequence functional diversity falls to validate functional diversity's use as a phenomenological indicator. To address this need, we must explore the currently lacking lagged relationships between functional diversity and ecosystem state to identify whether the former provides sufficient warning for management purposes and, if so, over what time horizon.

Lake environments have provided a peerless model for global change ecology as high‐resolution data are available from long‐term monitoring programmes for multiple sites around the world (Meinson et al., 2015). The plankton abundance data from these programmes are increasingly being supplemented with appropriate trait information, allowing lake communities to be classified and organized into discrete functional groupings within and across trophic levels (Kruk et al., 2011; Reynolds et al., 2002). However, more recently, there has been a shift towards continuous trait measures such as functional diversity (Abonyi, Ács, et al., 2018; Moody & Wilkinson, 2019; Ye et al., 2019) facilitated by the emergence of extensive trait databases (Hébert et al., 2016; Rimet & Druart, 2018) and guidance for trait‐based plankton research (Martini et al., 2021). Functional diversity metrics based on both literature‐average and study‐collected values have resultingly supported the predictions of biodiversity–ecosystem functioning theory (Tilman et al., 2014) by associating strongly with ecosystem functioning. For example, there is a positive correlation between functional diversity and phytoplankton biomass (Vogt et al., 2010) as well as a causal relationship with resource use efficiency (Ye et al., 2019), Similarly, zooplankton functional diversity correlates with trophic state (Moi et al., 2021; Moody & Wilkinson, 2019). However, each of these associations were only considered instantaneously when, in fact, lagged/leading associations may have been stronger. Explicit lagged effects are beginning to be considered more widely in system ecology (Gellner et al., 2020; Rastetter et al., 2021) and biodiversity research (Essl et al., 2015), but have not been considered during empirical biodiversity–functioning relationship assessments. Consequently, there are clear knowledge gaps regarding first, whether strong functional diversity–state associations are found consistently over time and among systems, and second, if functional diversity consistently changes prior to changes in commonly used state metrics such as density and community composition, allowing it to be a viable and generic leading indicator of ecosystem change. Using such phenomenological signals or forecasting techniques can act as a robust tool to test current scientific knowledge and improve ecological theory (Lewis et al., 2022). This is an underappreciated tool in ecology with functional ecology research a prime field to exploit these techniques due to the wealth of data and ecological theory underpinning it. For example, we expect functional diversity to satisfy the assumptions of a leading ecosystem indicator, but in the absence of any work (experimental or otherwise) on the topic, it is vital to challenge them prior to universally accepting functional diversity as an indicator of lake state change.

In this study, we use extensive plankton community datasets from five lakes around the world to assess whether phytoplankton and zooplankton functional diversity changes before, during or after changes in the state of the lake ecosystems. We quantify the usefulness of functional diversity as a monitoring tool for managers via cross correlation and novel empirical dynamic modelling at varying time lags, relative to state. A major hurdle to achieving this goal is that in complex, multivariate natural systems, co‐linearity and spurious correlations can inflate the strength of relationships while seasonality and measurement error can mask them. This uncertainty is compounded when considering lags which limits the ability for ecosystem managers to identify important relationships. We therefore exploit cutting edge empirical dynamic modelling techniques (Sugihara et al., 2012) specifically designed for analysing nonlinear dynamical systems using time‐series data, but introduce time lagged relationships following the approach of Ye et al. (2015). We demonstrate that functional diversity is weakly cross‐correlated with state, with associations often lake specific and synchronous, limiting functional diversity's usefulness as a management indicator. Causation assessment via convergent cross mapping (CCM; a.k.a. empirical dynamic modelling) yield a similar lack of consistency with bi‐directional causal relationships found, implying synchronicity between functional diversity and ecosystem state resulting from stronger extrinsic factors. However, unique dynamics present within the functional diversity time series highlight that due to the multidimensional nature of biodiversity, functional diversity still has value as an alternative measure of state even if it is not necessarily appropriate as a management indicator alone.

## 2 MATERIALS AND METHODS

### 2.1 Lake community data

Lake plankton density (individuals/ml) was compiled from five long‐term freshwater lake datasets curated by a range of government, university and not‐for‐profit sources: Lake Kasumigaura (Takamura et al., 2017; Takamura & Nakagawa, 2012), Lake Kinneret (Zohary, 2004), Lake Mendota (Carpenter et al., 2017a, 2017b), Windermere (Thackeray et al., 2015) and Lake Zurich (Pomati et al., 2020). This combination of lakes encompasses a range of longitudes, sizes and trophic regimes to provide a sufficiently broad representation of exploited freshwater lakes to test the constancy of plankton functional diversity and lake state associations (Table 1; Figure S1).

**TABLE 1:** Physical and limnological characteristics of the five lake communities assessed

Parameter	Lake Kasumigaura^a^	Lake Kinneret^b^ ^,^ ^c^	Lower Zurich^d^ ^,^ ^e^	Lake Mendota^f^ ^,^ ^g^	Windermere^h^
Lake area (km^2^)	168.0	168.7	65.0	39.6	250
Maximal depth (m)	7.0	45.0	136.0	25.3	42.0
Watershed area (km^2^)	1429.0	2730.0	1829.0	604.0	200.0
Mean retention time (days)	208.1	1533.0–3978.5 (increasing through time)	440.0	1642.5	100.0
Median annual surface water temperature (°C ± SE)	16.6 ± 0.37	23.1 ± 0.12	7.4 ± 0.11	10.8 ± 0.39	9.9 ± 0.29
Median annual nitrate (μgL^−1^ ± SE)	46.0 ± 9.48	103.6 ± 42.36	697.9 ± 4.00	252.3 ± 17.12	460.3 ± 10.44
Median annual total phosphorus (μgL^−1^ ± SE)	88.5 ± 1.57	16.7 ± 4.39	32.0 ± 0.99	102.0 ± 765.07	22.6 ± 0.37
Trophic status	Hyper eutrophic	Meso‐eutrophic	Mesotrophic	Eutrophic	Mesotrophic
Mixing regime	Polymictic (easily mixed due to its shallowness)	Monomictic	Dimictic	Monomictic	Monomictic

^a^ Havens et al. (2001).

^b^ Sukenik et al. (2014).

^c^ Zohary et al. (2014).

^d^ Fernández Castro et al. (2021).

^e^ Fiskal et al. (2019).

^f^ Duffy et al. (2018).

^g^ Gillon et al. (2015).

^h^ Moorhouse et al. (2018).

As the plankton datasets spanned multiple organizations, countries and sampling methodologies, we performed a standardization and quality control workflow. Unidentified and/or unnamed species were removed and if a species was not recorded on a sampling date, that species' density was assumed to be zero. The data were then averaged to mean density per month. To maintain the presence of rare species and better inform functional diversity/community estimates, we only further dropped species if their monthly time series consisted of more than 99% zeroes. A greater presence of zeroes than this prevented the completion of many downstream analyses. Any change in state of these five systems was then quantified from these standardized plankton density data using five metrics, each capturing a different dimension of state change (Table 2). Greater detail on each these metrics can be found in the Supplementary Information.

**TABLE 2:** A description of five system state metrics and an exemplary use in the literature

State metric	Calculation
Community composition (Community)	A dimension reduction of the plankton community comprising the first component of a principal component analysis across all species (Andersen et al., 2009; Hare & Mantua, 2000).
Total planktonic density (Density)	Summed densities of all plankton species (Kraemer et al., 2017). The sum is log transformed to linearize.
Fisher information (FI)	A measure of information content that can be adapted to assess the stability/order of a system (Fisher & Russell, 1922; Karunanithi et al., 2008). Decreasing *FI* implies decreasing system stability.
Multivariate index of variability (MVI)	The square root of the dominant eigenvalue of the covariance matrix of the plankton species timeseries (Brock & Carpenter, 2006) as a measure of system variance. Increasing MVI indicates increasing variability. The *MVI* is log transformed to linearize.
Trophic ratio (Z_P.ratio)	The ratio of zooplankton density to phytoplankton density, that is, the predator–prey relationship (Jeppesen et al., 2011; Warren & Gaston, 1992). The *Z_P.ratio* is log transformed to linearize.

### 2.2 Functional diversity

To underpin the functional diversity estimation, mean species‐level trait data were extracted from multiple published databases and articles (Arcifa et al., 2020; Borics et al., 2020; Hébert et al., 2016; Rimet & Druart, 2018). Here we consider traits as a measurable characteristic of an individual following Dawson et al. (2021). Traits were selected to encompass the three primary ecological axes relevant to phytoplankton (Litchman et al., 2013; Litchman & Klausmeier, 2008), namely resource acquisition, reproduction and predator avoidance (Table 3). Conversely, zooplankton traits are less available and so we followed the suggestions of Barnett et al. (2007) and Obertegger et al. (2011) to target the same ecological axes (Table 4).

**TABLE 3:** Functional traits of phytoplankton spanning the three primary ecological axes of interest

Guild	Ecological axis			Trait	Trait values
Phytoplankton	Resource acquisition	Reproduction	Predator defence	Cell length μm	≤10*
					>10‐25*
					>25‐100*
					>100*
				Surface area:volume ratio μm^2^/μm^3^	<0.5*
					>0.5‐1.0*
					>1.0‐5.0*
					>5.0‐10.0*
					>10.0*
				Organic carbon ratio	Numeric
				Trophy	Autotrophic/mixotrophic
				Nitrogen fixing	Yes/no
			Predator defence	Filamentous	Yes/no
		Reproduction		Mobility	None/flagellated/rapheated
				Colonial	Colonial
					Not colonial
				Siliceous	Yes/no

*Note*: Quantitative, qualitative and fuzzily coded values are possible, with fuzzy subcategories indicated by an *.

**TABLE 4:** Functional traits of zooplankton spanning the three primary ecological axes of interest

Guild	Ecological axis			Trait	Trait values
Zooplankton	Resource acquisition	Reproduction	Predator defence	Body length mm	≤0.3*
					>0.3‐0.9*
					>0.9‐1.5*
					>1.5*
				Trophic group	Herbivore
					Omnivore
					Carnivore
					Omnicarnivore
					Omniherbivore
				Feeding mode	*Bosmina*‐filtration/*Chydorus* filtration/*Daphnia*‐filtration/microfagous/raptorial/*Sida*‐filtration/suspension
		Reproduction		Reproductive mechanism	Sexual/asexual/cyclic parthenogenesis

*Note*: Qualitative and fuzzily coded values are possible, with fuzzy subcategories indicated by an *.

Trait classifications followed the original datasets but due to many lake monitoring programmes identifying plankton to the genus or family level, it was necessary to integrate multiple species' trait values into a single taxon value if functional diversity estimation was to be viable. This was achieved via a ‘fuzzy coding’ approach (Chevene et al., 1994) which involves the assignment of trait values representing the taxon's ‘affinity’ to a trait category based upon the variability of species values within it (i.e. the plasticity of the genus). A fuzzily coded trait matrix was therefore uniquely constructed for each lake and plankton guild (phytoplankton vs zooplankton) and from which we calculated a dissimilarity matrix (de Bello et al., 2021 see Supplementary Information) following the suggestions of Martini et al. (2021) for plankton communities. This dissimilarity matrix then underpinned three primary measures of functional diversity (reviewed by Mammola et al. (2021)): functional richness (*FRic*), functional dispersion (*FDis*) and functional evenness (*FEve*)—see Supplementary Information and Figure S2 for further details. All three measures were computed for each time point using the ‘*mFD*’ package (Magneville et al., 2022) with a reduced dimension space of 10 to escape generic errors caused during convex hull estimation at higher dimensions. This method therefore results in three functional diversity time series for each lake's phytoplankton and zooplankton guilds separately.

### 2.3 Associating system state and functional diversity

Prior to all analyses, system state and functional diversity metrics were scaled to zero mean and unit variance to ensure each shared the same level of magnitude and allow comparison between metrics and lakes. To capture the association between system state and functional diversity, and quantify whether functional diversity leads to changes in state, we performed cross‐correlations supplemented by permuted confidence intervals. Each functional diversity measure was cross correlated with each system state metric across a range of lags (from 0 to 60 months), and the observed Pearson correlation coefficient compared to a distribution of pseudorandom correlation coefficients. These coefficients were generated via permutation, where 10,000 surrogate functional diversity time series were constructed from a red/autocorrelated noise process informed by the observed data:

$$x_{1}=w_{1}$$

$$x_{t+1}=rx_{t}+{\left(1+r^{2}\right)}^{1/2}w_{t+1},t\geq 1$$

where *r* is the estimated autocorrelation coefficient of the observed time series as estimated by an ARIMA model (Ives et al., 2010) and *w* is a white noise process whose mean and variance equalled that of the observed time series. This red noise process consequently generates a surrogate series related to the observed time series but without the sudden changes in trend.

To limit the likelihood of spurious correlation, both the raw time series and permutations were made stationary prior to cross correlation by linear detrending. We then accounted for seasonality by additively decomposing the seasonal component of the original time series (i.e. the mean value for each month, across the length of the time series, standardized to sum to zero) and subtracting this estimate from the model residuals (Fortin et al., 2011; Zarnowitz & Ozyildirim, 2006).

The observed correlation coefficient at Lag0 and the strongest correlation (positive or negative) across lags were then compared to the permuted 2.5% and 97.5% quartiles to discriminate a stronger cross correlation than expected by serial dependence, and at which lags that may occur. If lags are negative and transgress these ‘confidence intervals’, functional diversity change occurs prior to changes in system state, whereas if lags are positive then functional diversity lags state change. Conversely, if the observed correlation resides within the 2.5% and 97.5% quartiles, then we consider functional diversity to not correlate significantly with system state.

### 2.4 Causality and CCM

To supplement this cross‐correlation approach and provide insight into the information content that functional diversity contains on system state, CCM was performed on the detrended time series (Sugihara et al., 2012). CCM allows the causal influence of one time series on another to be assessed by exploiting a hypothesized shared latent system (Chang et al., 2017—see Supplementary Information for details). The presence of forward and reverse causality—functional diversity causing system state and vice versa—for each lake and the optimal time delay (up to Lag60) of causation (Ye et al., 2015) was computed using the same permutation method as the cross‐correlation approach; if the observed cross map skill (analogous to correlation coefficient) between functional diversity and system state was greater than the 95th quartile of the distribution of cross map skills generated from 10,000 surrogate time series, then it was considered significant. Both forward and reverse causality require comparison as, unlike correlation, the strength of relationship depends on the direction of assessment, where strong cross map skills in both directions implies bi‐directional causality (Chang et al., 2017). All CCM analysis was performed using the ‘*rEDM*’ package (Park et al., 2021).

## 3 RESULTS

### 3.1 Distinct lake trends through time

**FIGURE 1:**
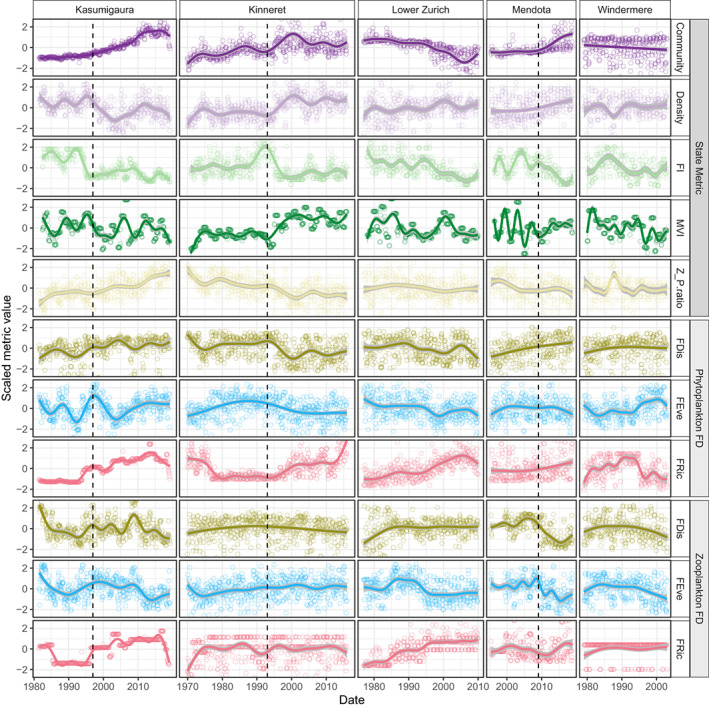
Smoothed time series of the five system state metrics, functional diversity of the two plankton trophic guilds and representation of environmental stressor in each of the five lakes. Smoothed trends are estimated by a generalized additive model of the metric through time and the vertical, dashed line represents literature reported regime shifts. Metric values are scaled to mean zero and unit variance.

We estimated three functional diversity metrics across two plankton trophic guilds in each of five lake monitoring datasets (Figure 1). Lake time series length varied in duration from 24 to 46 years, with a median length of 33 years. The final number of species that contributed to functional diversity estimates varied between lakes due to trait data limitations and longitudinal differences. Consequently, phytoplankton taxa record number ranged from 17 to 130 with a median of 79 taxa, whereas zooplankton records ranged from 4 to 31 taxa with a median of 22.

All lakes displayed turning points in their system state and functional diversity metrics, implying that over the course of monitoring these systems experienced some form of change in their communities (Figure 1). Most ecosystem state metrics changed simultaneously although Fisher information (*FI*) changed prior to changes in Community, Density and trophic ratio (*Z_P.ratio*), while the multivariate index of variability (*MVI*) displayed additional higher frequency fluctuations not identifiable in the other metrics. Functional diversity metrics also displayed unique trends depending on both the system and guild (phytoplankton or zooplankton), for example phytoplankton functional evenness (*phyFEve*) in Lake Kasumigaura displayed abrupt changes compared to other lakes, despite phytoplankton functional dispersion (*phyFDis*) being similar across lakes (Figure 1).

### 3.2 Synchronicity in both instantaneous and lagged cross‐correlation

**FIGURE 2:**
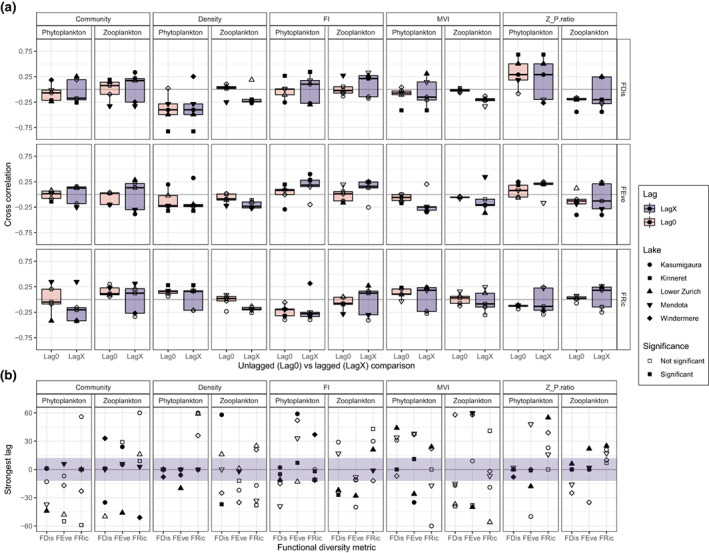
(a) Boxplots of cross correlations between each system state and functional diversity metric combination, estimated when functional diversity was unlagged relative to system state (Lag0) versus when it was lagged (LagX). These comparisons have then been stratified by functional diversity metric (FDis, FEve, FRic), state metric (Community, Density, Fisher information, Multivariate variance index, and Trophic ratio) and trophic level (phytoplankton vs. zooplankton). A filled point indicates that the mapping was in the strongest 5% of permuted mappings and is considered significant. LagX values represent the strongest cross map skill estimated separately for each lake and across all lags (−60 to +60 months). Consequently, lakes often displayed different strongest lags. (b) The spread of those lags across lakes for each functional diversity and system state metric combination. The dark band in panel b represents a ±1 year lag/lead, which, if a significant (filled) point is found, is considered a synchronous change between the functional diversity and state metric.

We consider correlations between functional diversity and system state in the form of both instantaneous/Lag0 correlations (i.e. between unlagged time series) and cross‐correlations (i.e. when one time series is lagged relative to the other). A strong instantaneous correlation would imply the functional dimension of biodiversity is related to state/functioning, whereas a strong cross‐correlation at a negative lag would suggests that functional diversity leads changes in state. However, we found correlations were inconsistent across all five of our lake systems (Figure 2, Figure S3), with each combination of functional diversity:system state varying in their proportion of significant correlations (Table S1). The phyFDis:Density relationship expressed the strongest average correlation at Lag0 (median ± SE: −0.40 ± 0.06) across all lakes and was significant in four of the five. The only other relationships of a similar magnitude were phyFDis:Z_P.ratio (0.29 ± 0.06) and phyFEve:Density (−0.22 ± 0.06) each of which was also significant in four of the five lakes. Conversely, zero significant correlations were observed between phyFRic:Z_P.ratio, zooFDis:MVI, zooFEve:MVI, zooFRic:Density, zooFRic:MVI and zooFRic:Z_P.ratio. When considered in isolation, Lake Kinneret (which showed the fastest and most distinct change in system state) displayed significant relationships for all but two combinations of indicators and phytoplankton functional diversity (phyFRic:Community and phyFRic:Z_P.ratio), whereas Windermere (whose state is most stationary) displayed no significant zooplankton relationships. Our results suggest that functional diversity is not universally correlated with system state in lake systems, but rather it is the unique dynamics/context that dictate(s) the strength of the relationship. One possible exception to this is phyFDis, which often strongly correlates with Density and Z_P.ratio.

When we considered lagged cross‐correlations (LagX), most relationships increased in average absolute correlation coefficient (Table S2), but only 13 of these relationships increased their proportion of significant correlations (14 others remained unchanged and three decreased). The phyFDis:Density relationship noted above emerged as universally shared (Figure 2, Figure S4), with phytoplankton functional diversity overall displaying stronger and more consistent relationships with system state than zooplankton functional diversity (with 35 significant relationships compared to zooplankton's 26; Figure 2, Figure S4). The optimal lag differed between the different functional diversity:system state relationships; for example, the phyFDis:Density relationship remaining strongest at lag0 (median correlation ± SE: −0.40 ± 0.08, median lag months ± SE: 0 ± 0.72), while phyFDis:Z_P.ratio (0.29 ± 0.08, 0 ± 0.78), phyFRic:FI (−0.28 ± 0.06, −2 ± 3.98) and phyFEve:MVI (−0.25 ± 0.05, 11 ± 6.86) combinations did appear equivalently strong but cluster within ±12 months of Lag0 (Figure 2d, Figure S5). These results indicate general synchrony between functional diversity and system state if the system expresses any relationship at all. Ultimately, cross‐correlations do not reveal clearly general leading nor lagging changes in functional diversity relative to system state but phyFDis is a good covariate of total planktonic density and trophic ratio at Lag0.

### 3.3 CCM reveals specific relationships of importance

**FIGURE 3:**
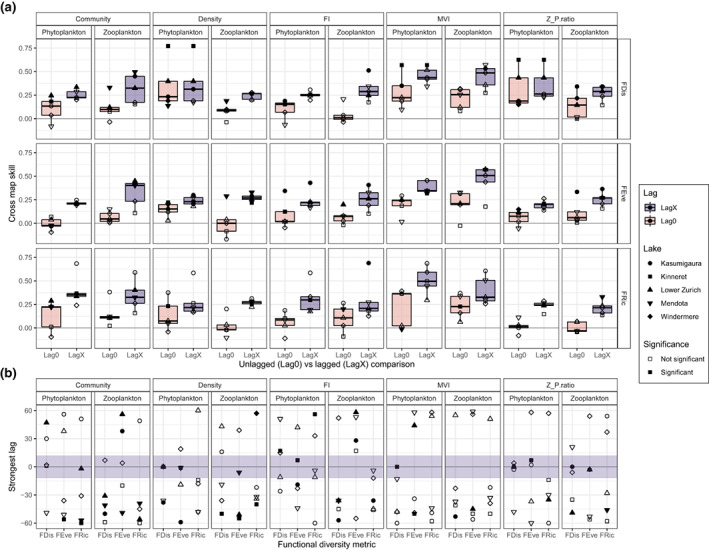
(a) Boxplots of cross mapping skills between each system state and functional diversity metric combination, estimated when functional diversity was unlagged relative to system state (Lag0) versus when it was lagged (LagX). These comparisons have then been stratified by functional diversity metric (FDis, FEve, FRic), state metric (Community, Density, Fisher information, Multivariate variance index and Trophic ratio) and trophic level (phytoplankton vs. zooplankton). A filled point indicates that the mapping was in the strongest 5% of permuted mappings and is considered significant. LagX values represent the strongest cross map skill estimated separately for each lake and across all lags (−60 to +60 months). Consequently, lakes often displayed different strongest lags. (b) The spread of those lags across lakes for each functional diversity and system state metric combination. The dark band in panel b represents a ±1 year lag/lead, which, if a significant (filled) point is found, is considered a synchronous change between the functional diversity and state metric.

When causal relationships were estimated using CCM, many of the observed weak cross‐correlations at Lag0 display causal forcing, although the more nuanced approach indicates that many of the previously identified correlations (Figure 2) may be spurious (Figure 3). For example, phyFDis:Density and phyFDis:Z_P.ratio mappings remain significant in all lakes but we found most mappings were within the permuted null distribution (Figure S6), suggesting no causal relationship between functional diversity and system state. The strongest average cross map skills were estimated for phyFRic:MVI (median ± SE: 0.37 ± 0.04), zooFDis:MVI (0.26 ± 0.02), phyFEve:MVI (0.24 ± 0.02) and phyFDis:Density (0.23 ± 0.05), although the variation was high between lakes (Figure S6; Table S4). Causality was also often found for the reverse relationship, where functional diversity maps system state, with the majority of the significant relationships expressing bidirectional causality (Figure S7; Tables S4 and S5)—that is, both functional diversity and system state influence one another, rather than one being a product of change in the other. This does not support strong causal relationships between functional diversity and system state, and suggests that the previously identified synchronous Lag0 correlations result from not the influence of diversity but from other extrinsic factors.

Interestingly, introducing lags improved the strength of causality by an average of 0.18 skill (implying that stronger causal relationships could be estimated from lagged data), yet did not increase the proportion of causal cross mappings (Figure 3, Figure S8). With lags, no relationship was significant in all lakes with only one significant in four of the five: zooFEve:Density (Figure S8, median skill ± SE: 0.28 ± 0.00; median lag months ± SE: −51 ± 8.29). Only the phyFRic:Density, phyFRic:MVI and zooFRic:MVI mappings remained universally non‐causal however. The optimal lag also differed between mappings with CCM displaying greater variation than that of the cross‐correlations and less prevalence within ±12 months (Figure 3d, Table S6). In fact, an almost tri‐modal distribution of significant lags are identifiable (Figure S9). It is also worth noting that counter to the cross‐correlation assessment, zooplankton functional diversity had a larger proportion of significant mappings than phytoplankton (37% vs. 27%).

**FIGURE 4:**
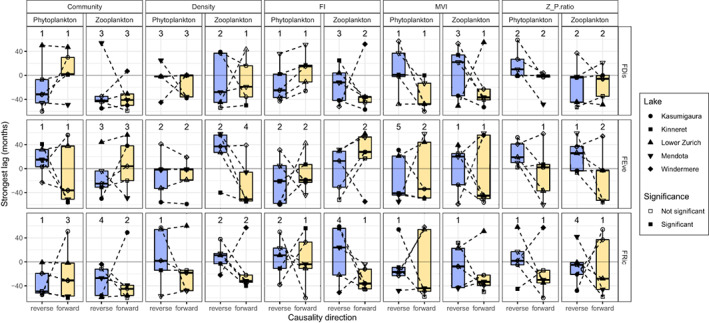
Boxplots of the paired lags between forward and reverse causal estimates for each functional diversity:system state combination. These comparisons have then been stratified by functional diversity metric (FDis, FEve, FRic), state metric (Community, Density, Fisher information, Multivariate variance index and Trophic ratio) and trophic level (phytoplankton vs. zooplankton). Filled points represent a significant causal relationship and the reported value is the number of significant mappings (out of five). Dashed lines link the two paired estimates (forward and reverse mappings within a lake). If one of these pairing lines crosses the grey, central lag line, then one of the metrics has a delayed impact and exerts sufficient causation on the other that synchronicity may occur. Variable directions in line/a flat line across all lakes can be interpreted that both metrics have equivalent causal delays upon each other.

Figure 4 presents the relative direction of causality between functional diversity and system state using the strongest lag as a method to disentangle forward causality from bidirectional. Using the zooFDis:Density relationship as an example (Figure 4a, second column), one forward causality assessment (zooFDis ‘causing’ Density) was significantly stronger than the permuted distribution (Kinneret) versus two reverse assessments (Density ‘causing’ zooFDis: Kasumigaura and Lower Zurich), but Kasumigaura's reverse assessment occurred at positive lags, whereas the forward assessment occurred at negative lags. This crossing of the central 0 month line of the dashed pairing line indicates that zooFDis' information leads Density's and that the influence of zooFDis synchronizes the two measurements. The opposite is true for Lower Zurich, whereas the flat line in Kinneret and Mendota suggest equal influence. If all lakes are considered together, then multiple crossing of pairing lines suggests no consistent causal relationship. Therefore, overall, considering the relative direction of causality between functional diversity and system state irrespective of significance, strong overlaps were identifiable between forward and reverse mappings for the majority of associations (Figure 4, Figure S10; Tables S7 and S8). The exceptions to this trend included FI leading phyFDis (Figure 4‐top row) and both phytoplankton and zooplankton FEve (Figure 4‐middle row). The reverse was true with Z_P.ratio being led by all but zooFDis and zooFRic, and Density being led by both phytoplankton and zooplankton FRic (Figure 4‐bottom row). These results imply functional diversity and system state are not strongly causally related, but both contain equivalent information on each other, with no consistent leading or lagging causality (Tables S5 and S6). Thus, most relationships are synchronous and support the overall cross‐correlation assessment. The FDis and Density relationship is identified as the most robust correlation and cross mapping however, and therefore represents the one strong functional diversity:system state association across lakes and trophic levels.

## 4 DISCUSSION

Here, we provide the first insights into how planktonic functional diversity estimated from literature average trait values temporally change relative to abundance‐based metrics of system state. Previous work has shown that functional diversity is related to system functioning, but we go beyond this by highlighting how considering the sequence of phenomenological signals is critical to understanding which dimensions of biodiversity are most sensitive to change. We find that simple correlative techniques described no or very weak synchronous relationships between functional diversity and state, an unexpected finding which is not substantially improved when lags between time series are considered. However, phytoplankton functional dispersion (*phyFDis*) was universally related with total planktonic density (*Density*) albeit synchronously. Conversely, bi‐directional causality was relatively prevalent between functional diversity and state when the complex lagged relationship is disentangled using CCM, although Fisher Information (*FI*) often led functional diversity. In both approaches, individual lakes expressed unique association strengths which limits our ability to make general conclusions on the use of functional diversity as a warning of ecosystem change. Functional diversity appears not to change consistently prior to abundance‐based system state metrics overall and so is impractical for use by managers as a pre‐emptive indicator of state change. It does however display unique dynamics distinct from state that may indicate their use as an alternative measure rather than a leading indicator.

### 4.1 Functional diversity relationships and time lags

The lack of state change pre‐emption by functional diversity conflicts with the assumption that trait information changes prior to changes in abundance (Clements & Ozgul, 2016; Williams et al., 2021). While some lagged relationships are identified by CCM for certain zooplankton functional diversity metrics, most relationships are strongest within ±12 months. Little information is available on the required time for managers to intervene to successfully mitigate state change, although restoration ecology indicates that most successful interventions require year to decade scales (Walker et al., 2014). There consequently appears insufficient time for action by ecosystem managers to revert oncoming state changes. This minimizes the usefulness of functional diversity as an early management tool.

This finding consequently highlights the general need to explicitly consider continuous time lags when considering functional traits. There are arguments that lags have been explored in functional ecology, but the majority focus on sub‐setting a time series in to pre−/post−/during‐disturbance periods (e.g. Boucek & Rehage, 2014; Uezu & Metzger, 2016) rather than in a continuous fashion as we have here. Our approach exploits the maximal information content of available across the time series while controlling for possible spurious associations to clarify conceptual assumptions. Having an inclination of ‘when’ system changes occur in addition to ‘whether’ they change is vital for intervention or preparation for functioning changes, and therefore is a necessary consideration for time‐series analyses.

Similarly, the absence of strong functional diversity–state relationships was unexpected considering the bulk of literature reconciling ecosystem state with the taxonomic and functional dimensions of biodiversity, both in planktonic (Abonyi, Horváth, et al., 2018; Moody & Wilkinson, 2019; Ye et al., 2019) and non‐planktonic communities (Cadotte et al., 2011; Díaz & Cabido, 2001; Gagic et al., 2015). However, it is not a universally identified relationship, with multivariate functional diversity measures failing to predict alpine biomass production (Zhu et al., 2016), the strength of relationship varying with disturbance in stream plant communities (Biswas & Mallik, 2011), and tree carbon stocks responding uniquely to individual forests' functional diversity (Ruiz‐Jaen & Potvin, 2010). Crucially, it is in controlled experiments that strong, consistent relationships are found, whereas observational studies are more variable. This is due to abiotic and biotic interactions filtering the taxa present at any moment in time to a ‘realized’ level of biodiversity that differs to the ‘true’/initial diversity examined in experiments (Hagan et al., 2021). We identify similar ambiguity here, with distinct relationships for individual lakes, each of which is known to be experiencing different levels of external stress. For example, Lake Kasumigaura and Lake Kinneret are considered to have undergone a regime shift in the late 1990s (Fukushima & Arai, 2015) and mid‐1990s (Roelke et al., 2007) respectively, whereas Windermere is relatively stable. Our system state metrics identify those two rapid regime changes and exhibit the strongest associations with functional diversity. Lake Kinneret in particular displays a sudden but relatively brief change in all its state metrics which is mirrored in its phytoplanktonic functional dispersion (*phyFDis)*.

It is likely the rapid change in state is the driver of the observed strong correlations between functional diversity and system state in Kinneret compared to the others, where the magnitude of change enforces an instantaneous shift in all the metrics we explored. This is supported by our paired CCMs where the lagging variable displays positive lags and the leading variable displays negative lags. When strong forcing is applied to a coupled system, the phenomenon of generalized synchrony can occur (Rulkov et al., 1995) as one system component exerts sufficiently strong causation on another that it brings them in to alignment/synchrony. Therefore, while synchronicity may be visible at short timescales, the leading variable is in fact exerting strong causality to synchronize the two (Ye et al., 2015). In ecology, the Moran effect (Moran, 1953) describes the phenomenon at macrospatial scales, with regime shifts acting as temporal analogues (Wernberg et al., 2013). The ubiquitous association in Lake Kinneret matches these examples as the synchrony strengthens for the short periods during the regime transition to improve the overall correlation. This implies that, while functional diversity's relationship is system specific, during regime transitions strong changes can be identified alongside typical system state measures, but does not pre‐empt them at management relevant timescales.

### 4.2 CCM, time lags and complex time series

The differences between CCM and cross‐correlation in characterizing the overall relationship between system state and functional diversity support the work of Sugihara et al. (2012) who show that traditional regression methods are unable to accurately identify complex associations between related ecological time series. Chang et al. (2022) also identified chained feedback effects in environmental driver–phytoplankton networks using the technique. Indeed, while non‐linear mappings revealed fewer stronger‐than‐null relationships than the correlative approach, CCM highlights the insightful and non‐spurious relationships of causal value. We find agreement that FDis is linked with total plankton density and that—when causation is present—typically both system state and functional diversity exert equal effects upon each other. In this regard, we consider the two measures as changing together, possibly in response to an unmeasured environmental variable, despite the strongest cross mappings occurring at negative lags. However, time delay effects are evident for certain metrics, particularly those involving FI. FI has previously been suggested to pre‐empt regime shifts in long time series (Ahmad et al., 2016; Cabezas et al., 2010; Spanbauer et al., 2014), where decreasing FI indicates decreasing stability of the system. There has been no extensive assessment of FI's capability in natural environments but, qualitatively, FI appears to change trajectory prior to each major turn point in the lakes explored in this study and can cross map/‘cause’ change in functional diversity.

This work highlights the practicality of CCM using the tools developed by Sugihara et al. (2012) and Ye et al. (2015). By reporting time lagged relationships and identifying broad stroke synchronicity, we hope that managers can exploit and interpret CCM outputs as part of their management toolbox. Indeed, there is growing encouragement for the consideration of time lags across functional (Lenoir et al., 2022) and conservation (Watts et al., 2020) ecology and we advocate CCM as one appropriate method of circumnavigating the complicated considerations this encouragement requires.

### 4.3 Lake‐specific considerations

One key difference between studies that may limit our identification of the expected strong associations between functional diversity and state is the length of time series. We find more significant relationships in the longer time series (Lake Kasumigaura, Kinneret and Zurich) than the short (Lake Mendota and Windermere). While this provides more data points for both correlation and CCM, our conservative approach of detrending and referencing an autocorrelated, permuted null distribution mitigates the likelihood of spurious correlations resulting from the shared system and larger datasets. As a result, we believe our results are valid.

The ability of functional diversity to pre‐empt system change may also be hampered by the quality of estimates from literature average values. Hutchinson's paradox (Hutchinson, 1961) highlights the high niche overlap of many planktonic species and resulting similarity in many routinely measured traits. This results in a community consisting of many functionally similar species when quantified from traits such as cell length or nitrogen‐fixing ability. It was this rationale that led to the development of Reynolds' phytoplankton functional groupings (Reynolds et al., 2002) to circumvent this apparent niche overlap and may indicate a weakness of the continuous functional diversity approach we applied here. While this complication is magnified by the lack of system specific trait information, it is our belief we were able to identify sufficiently distinct diversity trends and relationships using the proposed average‐trait framework of Martini et al. (2021) to validate the approach.

The use of average trait values is further validated by our understanding that the complex, multi‐trophic interactions occurring in these diverse lake communities minimize competitive exclusion and facilitate species with overlapping niches (Albert et al., 2021; Brose & Hillebrand, 2016). We see differences in the two plankton trophic levels' diversity but none sufficiently consistent to describe a universal pattern of top‐down/bottom‐up control. Strength of phytoplankton–zooplankton trophic coupling does vary with the degree of lake oligotrophication (Bernat et al., 2020; Carney & Elser, 1990; Dong et al., 2021) and presumably the lack of consistency results from the variable importance of phytoplankton versus zooplankton guilds in structuring the lake community. Zooplankton is particularly important in Lake Mendota for example, where the appearance of the invasive spiny water flea (*Bythotrephes longimanus*) (Walsh et al., 2017) decimated the zooplankton assemblage and initiated a trophic cascade towards a turbid, phytoplankton‐dominated community. This importance is identified in both our correlative and causality assessments, with minimal phytoplankton functional diversity associations with Mendota's system state compared to the improved pre‐emptive performance of certain zooplankton metrics.

A benefit of using lake systems is that due to their socio‐economic importance and long‐term monitoring, the influence of many environmental drivers is well reported and understood. We have not considered them explicitly here as we are solely focussing on the relationship between phenomenological measures rather than attempting to identify mechanistic drivers. However, typology and nutrient status may also be influencing the variable association strength between functional diversity and system state across lakes. For example, Lake Kasumigaura dynamics are specifically driven by nitrate concentrations (Matsuzaki et al., 2018), whereas temperature and phosphorus are more impactful in the Lower Zurich (Pomati et al., 2012) and the relative importance of nutrients in Lake Kinneret has shifted from phosphorus to nitrate during the time period assessed here (Gophen et al., 1999). There is therefore no unifying driver of the dynamics and associations we report and the associations identified are solely phenomenological signals. Consequently, when performing functional diversity assessments in or across lake ecosystems, it appears necessary to consider each system independently within its own context rather than attempt generalizations. This supports the suggestions of others that lakes consistently display unique dynamics and conditions (Adrian et al., 2009). We do stress that environmental variables are the drivers of trends we see in both functional diversity and state through time, and thus are key targets for any management intervention, but trait‐based indicators are best suited for local level representations of biodiversity.

The use of lakes for functional diversity research may also avoid previous concerns that our understanding of the mechanistic relationship between biodiversity and ecosystem functioning stems from field experiments where biodiversity effects can only be considered as ‘local’ (Hagan et al., 2021; Thompson et al., 2021). Island habitats have previously been considered as key study systems to assess the impacts of biodiversity due to their defined taxa pools matching the assumptions of much biodiversity–ecosystem functioning conceptual research (Kardol et al., 2018). Lakes may be considered biogeographically insular (MacDonald et al., 2018) with dispersal between neighbours restricted compared to the terrestrial environments underpinning much of our understanding of biodiversity–functioning relationships. Thus, the context surrounding lake plankton functional diversity may better represent the theoretical local level effects of biodiversity.

## 5 CONCLUSION

To conclude, the synchronous association between functional diversity and system state conflicts with the conceptual mechanistic relationship between biodiversity and ecosystem functioning. Most likely, any delayed impacts of functional diversity on our selected state measures are insufficiently long to warrant the use of functional diversity as an early indicator of ecosystem change, although the system‐specific dynamics of the functional metrics do sometimes yield unique dynamics not seen in the state measures. The relationship between functional diversity and ecosystem state will ultimately depend on the combination of environmental stressors, traits present and taxa interactions, which together potentially mask the overall relationship or highlight how each system is unique. The interpretation of functional diversity measures across systems therefore requires caution. Trait information is still vital to support our understanding of total biodiversity change, but dimensionally reduced trait measures like functional diversity are less informative than other abundance‐based phenomenological measures (such as FI) to ecosystem managers.

## 6 CONFLICT OF INTEREST

All authors declare no conflict of interest.

## Supporting information


Data S1

## Data Availability

Lake Kinneret and Lake Kasumigaura data are available on request, with all other data publicly available and referenced throughout. The public data that support the findings of this study are openly available in the EDI data portal at https://doi.org/10.6073/pasta/364622a6632f857289f9abc6a99d3ae7 and https://doi.org/10.6073/pasta/6fc6015c620056034512fde089d50c27, the NERC Environmental Information Data Centre at https://doi.org/10.5285/1de49dab‐c36e‐4700‐8b15‐93a639ae4d55, and the Eawag Research Data Institutional Repository at https://doi.org/10.25678/00039Z. All code for analysis is available in the Zenodo record (https://doi.org/10.5281/zenodo.7180270) and the associated GitHub repository (https://github.com/duncanobrien/plankton‐FD).

## References

[gcb16485-bib-0001] Abonyi, A. , Ács, É. , Hidas, A. , Grigorszky, I. , Várbíró, G. , Borics, G. , & Kiss, K. T. (2018). Functional diversity of phytoplankton highlights long‐term gradual regime shift in the middle section of the Danube river due to global warming, human impacts and oligotrophication. Freshwater Biology, 63, 456–472.

[gcb16485-bib-0002] Abonyi, A. , Horváth, Z. , & Ptacnik, R. (2018). Functional richness outperforms taxonomic richness in predicting ecosystem functioning in natural phytoplankton communities. Freshwater Biology, 63, 178–186.

[gcb16485-bib-0003] Adrian, R. , O'Reilly, C. M. , Zagarese, H. , Baines, S. B. , Hessen, D. O. , Keller, W. , Livingstone, D. M. , Sommaruga, R. , Straile, D. , Van Donk, E. , Weyhenmeyer, G. A. , & Winder, M. (2009). Lakes as sentinels of climate change. Limnology and Oceanography, 54, 2283–2297. 20396409 10.4319/lo.2009.54.6_part_2.2283 PMC2854826

[gcb16485-bib-0004] Ahmad, N. , Derrible, S. , Eason, T. , & Cabezas, H. (2016). Using Fisher information to track stability in multivariate systems. Royal Society Open Science, 3, 160582. 28018650 10.1098/rsos.160582 PMC5180148

[gcb16485-bib-0005] Albert, G. , Gauzens, B. , Loreau, M. , Wang, S. , & Brose, U. (2021). The hidden role of multi‐trophic interactions in driving diversity–productivity relationships. Ecology Letters, 25, 405–415. 34846785 10.1111/ele.13935

[gcb16485-bib-0006] Andersen, T. , Carstensen, J. , Hernández‐García, E. , & Duarte, C. M. (2009). Ecological thresholds and regime shifts: approaches to identification. Trends in Ecology and Evolution, 24, 49–57. 18952317 10.1016/j.tree.2008.07.014

[gcb16485-bib-0007] Arcifa, M. S. , de Souza, B. B. , de Morais‐Junior, C. S. , & Bruno, C. G. C. (2020). Functional groups of rotifers and an exotic species in a tropical shallow lake. Scientific Reports, 10, 14698. 32895424 10.1038/s41598-020-71778-1 PMC7477540

[gcb16485-bib-0008] Baker, N. J. , Pilotto, F. , Haubrock, P. J. , Beudert, B. , & Haase, P. (2021). Multidecadal changes in functional diversity lag behind the recovery of taxonomic diversity. Ecology and Evolution, 11, 17471–17484. 34938522 10.1002/ece3.8381 PMC8668763

[gcb16485-bib-0009] Barnett, A. J. , Finlay, K. , & Beisner, B. E. (2007). Functional diversity of crustacean zooplankton communities: towards a trait‐based classification. Freshwater Biology, 52, 796–813.

[gcb16485-bib-0010] Bernat, G. , Boross, N. , Somogyi, B. , Voros, L. , G‐Toth, L. , & Boros, G. (2020). Oligotrophication of Lake Balaton over a 20‐year period and its implications for the relationship between phytoplankton and zooplankton biomass. Hydrobiologia, 847, 3999–4013.

[gcb16485-bib-0011] Biswas, S. R. , & Mallik, A. U. (2011). Species diversity and functional diversity relationship varies with disturbance intensity. Ecosphere, 2(4), 1–10. 10.1890/ES10-00206.1

[gcb16485-bib-0012] Borics, G. , B‐Béres, V. , Bácsi, I. , Lukács, B. A. , T‐Krasznai, E. , Botta‐Dukát, Z. , & Várbíró, G. (2020). Trait convergence and trait divergence in lake phytoplankton reflect community assembly rules. Scientific Reports, 10, 19599. 33177646 10.1038/s41598-020-76645-7 PMC7658209

[gcb16485-bib-0013] Boucek, R. E. , & Rehage, J. S. (2014). Climate extremes drive changes in functional community structure. Global Change Biology, 20, 1821–1831. 24733813 10.1111/gcb.12574

[gcb16485-bib-0014] Brock, W. , & Carpenter, S. (2006). Variance as a leading indicator of regime shift in ecosystem services. Ecology and Society, 11, 9.

[gcb16485-bib-0015] Brose, U. , & Hillebrand, H. (2016). Biodiversity and ecosystem functioning in dynamic landscapes. Philosophical Transactions of the Royal Society B: Biological Sciences, 371, 20150267. 10.1098/rstb.2015.0267 PMC4843689 27114570

[gcb16485-bib-0016] Bullock, J. M. , Fuentes‐Montemayor, E. , McCarthy, B. , Park, K. , Hails, R. S. , Woodcock, B. A. , Watts, K. , Corstanje, R. , & Harris, J. (2022). Future restoration should enhance ecological complexity and emergent properties at multiple scales. Ecography, 2022. 10.1111/ecog.05780

[gcb16485-bib-0017] Cabezas, H. , Campbell, D. , Eason, T. , Garmestani, A. S. , Heberling, M. T. , Hopton, M. E. , Templeton, J. , White, D. , Zanowick, M. , & Sparks, R. T. (2010). In M. T. Heberling & M. E. Hopton (Eds.), San Luis Basin sustainability metrics project: A methodology for evaluating regional sustainability (pp. 119–136). The United States Environmental Protection Agency.

[gcb16485-bib-0018] Cadotte, M. W. , Carscadden, K. , & Mirotchnick, N. (2011). Beyond species: functional diversity and the maintenance of ecological processes and services. Journal of Applied Ecology, 48, 1079–1087.

[gcb16485-bib-0021] Cardinale, B. J. , Nelson, K. , & Palmer, M. A. (2000). Linking species diversity to the functioning of ecosystems: On the importance of environmental context. Oikos, 91, 175–183.

[gcb16485-bib-0022] Cardinale, B. J. , Srivastava, D. S. , Emmett Duffy, J. , Wright, J. P. , Downing, A. L. , Sankaran, M. , & Jouseau, C. (2006). Effects of biodiversity on the functioning of trophic groups and ecosystems. Nature, 443, 989–992. 17066035 10.1038/nature05202

[gcb16485-bib-0023] Carney, H. J. , & Elser, J. J. (1990). Strength of zooplankton‐phytoplankton coupling in relation to lake trophic state. In M. M. Tilzer & C. Serruya (Eds.), Large lakes: Ecological structure and function (pp. 615–631). Springer Berlin Heidelberg.

[gcb16485-bib-0024] Carpenter, S. , Kitchell, J. , Cole, J. , & Pace, M. (2017a). Cascade project at North Temperate Lakes LTER core data phytoplankton 1984–2015 ver 4. Environmental Data Initiative.

[gcb16485-bib-0025] Carpenter, S. , Kitchell, J. , Cole, J. , & Pace, M. (2017b). Cascade project at North Temperate Lakes LTER core data zooplankton 1984–2016 ver 4. Environmental Data Initiative.

[gcb16485-bib-0026] Chang, C.‐W. , Miki, T. , Ye, H. , Souissi, S. , Adrian, R. , Anneville, O. , Agasild, H. , Ban, S. , Be'eri‐Shlevin, Y. , Chiang, Y. R. , Feuchtmayr, H. , Gal, G. , Ichise, S. , Kagami, M. , Kumagai, M. , Liu, X. , Matsuzaki, S. S. , Manca, M. M. , Nõges, P. , … Hsieh, C. H. (2022). Causal networks of phytoplankton diversity and biomass are modulated by environmental context. Nature Communications, 13, 1140. 10.1038/s41467-022-28761-3 PMC8894464 35241667

[gcb16485-bib-0027] Chang, C.‐W. , Ushio, M. , & Hsieh, C. (2017). Empirical dynamic modeling for beginners. Ecological Research, 32, 785–796.

[gcb16485-bib-0028] Chevene, F. , Doleadec, S. , & Chessel, D. (1994). A fuzzy coding approach for the analysis of long‐term ecological data. Freshwater Biology, 31, 295–309.

[gcb16485-bib-0029] Clements, C. F. , & Ozgul, A. (2016). Including trait‐based early warning signals helps predict population collapse. Nature Communications, 7, 10984. 10.1038/ncomms10984 PMC4820807 27009968

[gcb16485-bib-0030] Dale, V. H. , & Beyeler, S. C. (2001). Challenges in the development and use of ecological indicators. Ecological Indicators, 1, 3–10.

[gcb16485-bib-0031] Dawson, S. P. , Carmona, C. , González‐Suárez, M. , Jönsson, M. , Chichorro, F. , Mallen‐Cooper, M. , Melero, Y. , Moor, H. , Simaika, J. P. , & Duthie, A. B. (2021). The traits of “trait ecologists”: An analysis of the use of trait and functional trait terminology. Ecology and Evolution, 11, 16434–16445. 34938447 10.1002/ece3.8321 PMC8668725

[gcb16485-bib-0032] de Bello, F. , Botta‐Dukát, Z. , Lepš, J. , & Fibich, P. (2021). Towards a more balanced combination of multiple traits when computing functional differences between species. Methods in Ecology and Evolution, 12, 443–448.

[gcb16485-bib-0033] de Bello, F. , Lavorel, S. , Díaz, S. , Harrington, R. , Cornelissen, J. H. C. , Bardgett, R. D. , Berg, M. P. , Cipriotti, P. , Feld, C. K. , Hering, D. , da Silva, P. M. , Potts, S. G. , Sandin, L. , Sousa, J. P. , Storkey, J. , Wardle, D. A. , & Harrison, P. A. (2010). Towards an assessment of multiple ecosystem processes and services via functional traits. Biodiversity and Conservation, 19, 2873–2893.

[gcb16485-bib-0034] Díaz, S. , & Cabido, M. (2001). Vive la différence: plant functional diversity matters to ecosystem processes. Trends in Ecology and Evolution, 16, 646–655.

[gcb16485-bib-0035] Dong, K. X. , Kvile, K. O. , Stenseth, N. C. , & Stige, L. C. (2021). Associations between timing and magnitude of spring blooms and zooplankton dynamics in the southwestern Barents Sea. Marine Ecology Progress Series, 668, 57–72.

[gcb16485-bib-0501] Duffy, C. , Dugan, H. , & Hanson, P. (2018). The age of water and carbon in lake‐catchments: A simple dynamical model: Age of water and carbon in lake‐catchments. Limnology and Oceanography Letters, 3, 236–245. 10.1002/lol2.10070

[gcb16485-bib-0036] Duffy, J. E. , Godwin, C. M. , & Cardinale, B. J. (2017). Biodiversity effects in the wild are common and as strong as key drivers of productivity. Nature, 549, 261–264. 28869964 10.1038/nature23886

[gcb16485-bib-0037] Edwards, F. A. , Edwards, D. P. , Larsen, T. H. , Hsu, W. W. , Benedick, S. , Chung, A. , Vun Khen, C. , Wilcove, D. S. , & Hamer, K. C. (2014). Does logging and forest conversion to oil palm agriculture alter functional diversity in a biodiversity hotspot? Animal Conservation, 17, 163–173. 25821399 10.1111/acv.12074 PMC4372061

[gcb16485-bib-0038] Essl, F. , Dullinger, S. , Rabitsch, W. , Hulme, P. E. , Pyšek, P. , Wilson, J. R. U. , & Richardson, D. M. (2015). Historical legacies accumulate to shape future biodiversity in an era of rapid global change. Diversity and Distributions, 21, 534–547.

[gcb16485-bib-0502] Fernández Castro, B. , Sepúlveda Steiner, O. , Knapp, D. , Posch, T. , Bouffard, D. , & Wüest, A. (2021). Inhibited vertical mixing and seasonal persistence of a thin cyanobacterial layer in a stratified lake. Aquatic Sciences, 83(2), 38. 10.1007/s00027-021-00785-9

[gcb16485-bib-0039] Fisher, R. A. , & Russell, E. J. (1922). On the mathematical foundations of theoretical statistics. Philosophical Transactions of the Royal Society of London Series A: Containing Papers of a Mathematical or Physical Character, 222, 309–368.

[gcb16485-bib-0503] Fiskal, A. , Deng, L. , Michel, A. , Eickenbusch, P. , Han, X. , Lagostina, L. , Zhu, R. , Sander, M. , Schroth, M. H. , Bernasconi, S. M. , Dubois, N. , & Lever, M. A. (2019). Effects of eutrophication on sedimentary organic carbon cycling in five temperate lakes. Biogeosciences, 16(19), 3725–3746. 10.5194/bg-16-3725-2019

[gcb16485-bib-0504] Fortin, N. , Lemieux, T. , & Firpo, S. (2011). Chapter 1—Decomposition methods in economics. In O. Ashenfelter & D. Card (Eds.), Handbook of labor economics (Vol. 4, pp. 1–102). Elsevier. 10.1016/S0169-7218(11)00407-2

[gcb16485-bib-0040] Fukami, T. , Martijn Bezemer, T. , Mortimer, S. R. , & van der Putten, W. H. (2005). Species divergence and trait convergence in experimental plant community assembly. Ecology Letters, 8, 1283–1290.

[gcb16485-bib-0041] Fukushima, T. , & Arai, H. (2015). Regime shifts observed in Lake Kasumigaura, a large shallow lake in Japan: Analysis of a 40‐year limnological record. Lakes & Reservoirs: Research & Management, 20, 54–68. 10.1111/lre.12085

[gcb16485-bib-0042] Gagic, V. , Bartomeus, I. , Jonsson, T. , Taylor, A. , Winqvist, C. , Fischer, C. , Slade, E. M. , Steffan‐Dewenter, I. , Emmerson, M. , Potts, S. G. , Tscharntke, T. , Weisser, W. , & Bommarco, R. (2015). Functional identity and diversity of animals predict ecosystem functioning better than species‐based indices. Proceedings of the Royal Society B: Biological Sciences, 282, 20142620. 10.1098/rspb.2014.2620 PMC4309003 25567651

[gcb16485-bib-0043] Gellner, G. , McCann, K. S. , & Greyson‐Gaito, C. (2020). The synergistic effects of interaction strength and lags on ecological stability. In Theoretical ecology (pp. 28–39). Oxford University Press.

[gcb16485-bib-0505] Gillon, S. , Booth, E. , & Rissman, A. (2015). Shifting drivers and static baselines in environmental governance: Challenges for improving and proving water quality outcomes. Regional Environmental Change, 16, 759–775. 10.1007/s10113-015-0787-0

[gcb16485-bib-0044] Gophen, M. , Smith, V. H. , Nishri, A. , & Threlkeld, S. T. (1999). Nitrogen deficiency, phosphorus sufficiency, and the invasion of Lake Kinneret, Israel, by the N_2_‐fixing cyanobacterium *Aphanizomenon ovalisporum* . Aquatic Sciences, 61, 293–306.

[gcb16485-bib-0046] Hagan, J. G. , Vanschoenwinkel, B. , & Gamfeldt, L. (2021). We should not necessarily expect positive relationships between biodiversity and ecosystem functioning in observational field data. Ecology Letters, 24, 2537–2548. 34532926 10.1111/ele.13874

[gcb16485-bib-0047] Hare, S. R. , & Mantua, N. J. (2000). Empirical evidence for North Pacific regime shifts in 1977 and 1989. Progress in Oceanography, 47, 103–145.

[gcb16485-bib-0048] Hastings, A. (2016). Timescales and the management of ecological systems. Proceedings of the National Academy of Sciences, 113, 14568. 10.1073/pnas.1604974113 PMC5187717 27729535

[gcb16485-bib-0506] Havens, K. E. , Fukushima, T. , Xie, P. , Iwakuma, T. , James, R. T. , Takamura, N. , Hanazato, T. , & Yamamoto, T. (2001). Nutrient dynamics and the eutrophication of shallow lakes Kasumigaura (Japan), Donghu (PR China), and Okeechobee (USA). Environmental Pollution, 111(2), 263–272. 10.1016/S0269-7491(00)00074-9 11202730

[gcb16485-bib-0049] Hébert, M.‐P. , Beisner, B. E. , & Maranger, R. (2016). A compilation of quantitative functional traits for marine and freshwater crustacean zooplankton. Ecology, 97, 1081. 28792594 10.1890/15-1275.1

[gcb16485-bib-0050] Hutchinson, G. E. (1961). The paradox of the plankton. The American Naturalist, 95, 137–145.

[gcb16485-bib-0051] Ives, A. R. , Abbott, K. C. , & Ziebarth, N. L. (2010). Analysis of ecological time series with ARMA(p,q) models. Ecology, 91, 858–871. 20426343 10.1890/09-0442.1

[gcb16485-bib-0052] Jeppesen, E. , Nõges, P. , Davidson, T. A. , Haberman, J. , Nõges, T. , Blank, K. , Lauridsen, T. L. , Søndergaard, M. , Sayer, C. , Laugaste, R. , Johansson, L. S. , Bjerring, R. , & Amsinck, S. L. (2011). Zooplankton as indicators in lakes: A scientific‐based plea for including zooplankton in the ecological quality assessment of lakes according to the European Water Framework Directive (WFD). Hydrobiologia, 676, 279.

[gcb16485-bib-0053] Kardol, P. , Fanin, N. , & Wardle, D. A. (2018). Long‐term effects of species loss on community properties across contrasting ecosystems. Nature, 557, 710–713. 29795345 10.1038/s41586-018-0138-7

[gcb16485-bib-0054] Karunanithi, A. T. , Cabezas, H. , Frieden, B. R. , & Pawlowski, C. W. (2008). Detection and assessment of ecosystem regime shifts from Fisher information. Ecology and Society, 13(1), 22.

[gcb16485-bib-0055] Kraemer, B. M. , Mehner, T. , & Adrian, R. (2017). Reconciling the opposing effects of warming on phytoplankton biomass in 188 large lakes. Scientific Reports, 7, 10762. 28883487 10.1038/s41598-017-11167-3 PMC5589843

[gcb16485-bib-0056] Kruk, C. , Peeters, E. T. H. M. , van Nes, E. H. , Huszar, V. L. M. , Costa, L. S. , & Scheffer, M. (2011). Phytoplankton community composition can be predicted best in terms of morphological groups. Limnology and Oceanography, 56, 110–118.

[gcb16485-bib-0058] Lenoir, J. , Gril, E. , Durrieu, S. , Horen, H. , Laslier, M. , Lembrechts, J. J. , Zellweger, F. , Alleaume, S. , Brasseur, B. , Buridant, J. , Dayal, K. , De Frenne, P. , Gallet‐Moron, E. , Marrec, R. , Meeussen, C. , Rocchini, D. , Van Meerbeek, K. , & Decocq, G. (2022). Unveil the unseen: Using LiDAR to capture time‐lag dynamics in the herbaceous layer of European temperate forests. Journal of Ecology, 110, 282–300.

[gcb16485-bib-0059] Lewis, A. S. L. , Rollinson, C. R. , Allyn, A. J. , Ashander, J. , Brodie, S. , Brookson, C. B. , Collins, E. , Dietze, M. C. , Gallinat, A. S. , Juvigny‐Khenafou, N. , Koren, G. , McGlinn, D. J. , Moustahfid, H. , Peters, J. A. , Record, N. R. , Robbins, C. J. , Tonkin, J. , & Wardle, G. M. (2022). The power of forecasts to advance ecological theory. Methods in Ecology and Evolution, 1–11. 10.1111/2041-210X.13955

[gcb16485-bib-0060] Litchman, E. , & Klausmeier, C. A. (2008). Trait‐based community ecology of phytoplankton. Annual Review of Ecology, Evolution, and Systematics, 39, 615–639.

[gcb16485-bib-0061] Litchman, E. , Ohman, M. D. , & Kiørboe, T. (2013). Trait‐based approaches to zooplankton communities. Journal of Plankton Research, 35, 473–484.

[gcb16485-bib-0062] Lyashevska, O. , & Farnsworth, K. D. (2012). How many dimensions of biodiversity do we need? Ecological Indicators, 18, 485–492.

[gcb16485-bib-0063] MacDonald, Z. G. , Anderson, I. D. , Acorn, J. H. , & Nielsen, S. E. (2018). The theory of island biogeography, the sample‐area effect, and the habitat diversity hypothesis: complementarity in a naturally fragmented landscape of lake islands. Journal of Biogeography, 45, 2730–2743.

[gcb16485-bib-0064] Magneville, C. , Loiseau, N. , Albouy, C. , Casajus, N. , Claverie, T. , Escalas, A. , Leprieur, F. , Maire, E. , Mouillot, D. , & Villéger, S. (2022). mFD: an R package to compute and illustrate the multiple facets of functional diversity. Ecography, 2022. 10.1111/ecog.05904

[gcb16485-bib-0065] Mammola, S. , Carmona, C. P. , Guillerme, T. , & Cardoso, P. (2021). Concepts and applications in functional diversity. Functional Ecology, 35, 1869–1885. 10.1111/1365-2435.13882

[gcb16485-bib-0066] Martini, S. , Larras, F. , Boyé, A. , Faure, E. , Aberle, N. , Archambault, P. , Bacouillard, L. , Beisner, B. E. , Bittner, L. , Castella, E. , Danger, M. , Gauthier, O. , Karp‐Boss, L. , Lombard, F. , Maps, F. , Stemmann, L. , Thiébaut, E. , Usseglio‐Polatera, P. , Vogt, M. , … Ayata, S.‐D. (2021). Functional trait‐based approaches as a common framework for aquatic ecologists. Limnology and Oceanography, 66, 965–994.

[gcb16485-bib-0067] Matsuzaki, S. S. , Suzuki, K. , Kadoya, T. , Nakagawa, M. , & Takamura, N. (2018). Bottom‐up linkages between primary production, zooplankton, and fish in a shallow, hypereutrophic lake. Ecology, 99, 2025–2036. 29884987 10.1002/ecy.2414

[gcb16485-bib-0068] McGill, B. J. , Enquist, B. J. , Weiher, E. , & Westoby, M. (2006). Rebuilding community ecology from functional traits. Trends in Ecology and Evolution, 21, 178–185. 16701083 10.1016/j.tree.2006.02.002

[gcb16485-bib-0069] Meinson, P. , Idrizaj, A. , Nõges, P. , Nõges, T. , & Laas, A. (2015). Continuous and high‐frequency measurements in limnology: History, applications, and future challenges. Environmental Reviews, 24, 52–62.

[gcb16485-bib-0070] Moi, D. A. , Romero, G. Q. , Jeppesen, E. , Kratina, P. , Alves, D. C. , Antiqueira, P. A. P. , Teixeira de Mello, F. , Figueiredo, B. R. , Bonecker, C. C. , Pires, A. P. , Braghin, L. S. , & Mormul, R. P. (2021). Regime shifts in a shallow lake over 12 years: Consequences for taxonomic and functional diversity, and ecosystem multifunctionality. Journal of Animal Ecology, 91, 551–565. 10.1111/1365-2656.13658 34954827

[gcb16485-bib-0071] Moody, E. K. , & Wilkinson, G. M. (2019). Functional shifts in lake zooplankton communities with hypereutrophication. Freshwater Biology, 64, 608–616.

[gcb16485-bib-0507] Moorhouse, H. L. , McGowan, S. , Taranu, Z. E. , Gregory‐Eaves, I. , Leavitt, P. R. , Jones, M. D. , Barker, P. , & Brayshaw, S. A. (2018). Regional versus local drivers of water quality in the Windermere catchment, Lake District, United Kingdom: The dominant influence of wastewater pollution over the past 200 years. Global Change Biology, 24(9), 4009–4022. 10.1111/gcb.14299 29749028

[gcb16485-bib-0072] Moran, P. A. P. (1953). The statistical analysis of the Canadian Lynx cycle. Australian Journal of Zoology, 1, 291–298.

[gcb16485-bib-0073] Mouillot, D. , Villéger, S. , Scherer‐Lorenzen, M. , & Mason, N. W. H. (2011). Functional structure of biological communities predicts ecosystem multifunctionality. PLoS ONE, 6, e17476. 21423747 10.1371/journal.pone.0017476 PMC3053366

[gcb16485-bib-0074] Naeem, S. , Prager, C. , Weeks, B. , Varga, A. , Flynn, D. F. B. , Griffin, K. , Muscarella, R. , Palmer, M. , Wood, S. , & Schuster, W. (2016). Biodiversity as a multidimensional construct: a review, framework and case study of herbivory's impact on plant biodiversity. Proceedings of the Royal Society B: Biological Sciences, 283, 20153005. 10.1098/rspb.2015.3005 PMC5204135 27928041

[gcb16485-bib-0075] Obertegger, U. , Smith, H. A. , Flaim, G. , & Wallace, R. L. (2011). Using the guild ratio to characterize pelagic rotifer communities. Hydrobiologia, 662, 157–162.

[gcb16485-bib-0508] Oliver, T. H. , Heard, M. S. , Isaac, N. J. B. , Roy, D. B. , Procter, D. , Eigenbrod, F. , Freckleton, R. , Hector, A. , Orme, C. D. L. , Petchey, O. L. , Proença, V. , Raffaelli, D. , Suttle, K. B. , Mace, G. M. , Martín‐López, B. , Woodcock, B. A. , & Bullock, J. M. (2015). Biodiversity and resilience of ecosystem functions. Trends in Ecology and Evolution, 30(11), 673–684. 10.1016/j.tree.2015.08.009 26437633

[gcb16485-bib-0076] Park, J. , Smith, C. , Sugihara, G. & Deyle, E. (2021). rEDM: Empirical Dynamic Modeling ('EDM'). R package version 1.13.1. https://CRAN.R‐project.org/package=rEDM

[gcb16485-bib-0078] Pomati, F. , Matthews, B. , Jokela, J. , Schildknecht, A. , & Ibelings, B. W. (2012). Effects of re‐oligotrophication and climate warming on plankton richness and community stability in a deep mesotrophic lake. Oikos, 121, 1317–1327.

[gcb16485-bib-0079] Pomati, F. , Shurin, J. B. , Andersen, K. H. , Tellenbach, C. , & Barton, A. D. (2020). Interacting temperature, nutrients and zooplankton grazing control phytoplankton size‐abundance relationships in eight Swiss lakes. Frontiers in Microbiology, 10, 3155. 32038586 10.3389/fmicb.2019.03155 PMC6987318

[gcb16485-bib-0080] Rastetter, E. B. , Ohman, M. D. , Elliott, K. J. , Rehage, J. S. , Rivera‐Monroy, V. H. , Boucek, R. E. , Castañeda‐Moya, E. , Danielson, T. M. , Gough, L. , Groffman, P. M. , Jackson, C. R. , Miniat, C. F. , & Shaver, G. R. (2021). Time lags: Insights from the U.S. Long Term Ecological Research Network. Ecosphere, 12, e03431.

[gcb16485-bib-0081] Regos, A. , Gagne, L. , Alcaraz‐Segura, D. , Honrado, J. P. , & Domínguez, J. (2019). Effects of species traits and environmental predictors on performance and transferability of ecological niche models. Scientific Reports, 9, 4221. 30862919 10.1038/s41598-019-40766-5 PMC6414724

[gcb16485-bib-0082] Reynolds, C. S. , Huszar, V. , Kruk, C. , Naselli‐Flores, L. , & Melo, S. (2002). Towards a functional classification of the freshwater phytoplankton. Journal of Plankton Research, 24, 417–428.

[gcb16485-bib-0083] Rimet, F. , & Druart, J.‐C. (2018). A trait database for phytoplankton of temperate lakes. Annales De Limnologie‐International Journal of Limnology, 54, 18. 10.1051/limn/2018009

[gcb16485-bib-0084] Rockström, J. , Steffen, W. , Noone, K. , Persson, Å. , Chapin, F. S. , Lambin, E. F. , Lenton, T. M. , Scheffer, M. , Folke, C. , Schellnhuber, H. J. , Nykvist, B. , de Wit, C. A. , Hughes, T. , van der Leeuw, S. , Rodhe, H. , Sörlin, S. , Snyder, P. K. , Costanza, R. , Svedin, U. , … Foley, J. A. (2009). A safe operating space for humanity. Nature, 461, 472–475. 19779433 10.1038/461472a

[gcb16485-bib-0085] Roelke, D. L. , Zohary, T. , Hambright, K. D. , & Montoya, J. V. (2007). Alternative states in the phytoplankton of Lake Kinneret, Israel (Sea of Galilee). Freshwater Biology, 52, 399–411.

[gcb16485-bib-0086] Ruiz‐Jaen, M. , & Potvin, C. (2010). Can we predict carbon stocks in tropical ecosystems from tree diversity? Comparing species and functional diversity in a plantation and a natural forest. New Phytologist, 189, 978–987. 20958305 10.1111/j.1469-8137.2010.03501.x

[gcb16485-bib-0087] Rulkov, N. F. , Sushchik, M. M. , Tsimring, L. S. , & Abarbanel, H. D. I. (1995). Generalized synchronization of chaos in directionally coupled chaotic systems. Physical Review E, 51, 980–994. 10.1103/physreve.51.980 9962737

[gcb16485-bib-0088] Spanbauer, T. L. , Allen, C. R. , Angeler, D. G. , Eason, T. , Fritz, S. C. , Garmestani, A. S. , Nash, K. L. , & Stone, J. R. (2014). Prolonged instability prior to a regime shift. PLoS ONE, 9, e108936. 25280010 10.1371/journal.pone.0108936 PMC4184814

[gcb16485-bib-0089] Sugihara, G. , May, R. , Ye, H. , Hsieh, C. , Deyle, E. , Fogarty, M. , & Munch, S. (2012). Detecting causality in complex ecosystems. Science, 338, 496–500. 22997134 10.1126/science.1227079

[gcb16485-bib-0509] Sukenik, A. , Zohary, T. , & Markel, D. (2014). The monitoring program. In T. Zohary , A. Sukenik , T. Berman , & A. Nishri (Eds.) Lake Kinneret: Aquatic ecology series (Vol. 6). Springer. 10.1007/978-94-017-8944-8_32

[gcb16485-bib-0090] Takamura, N. , & Nakagawa, M. (2012). Phytoplankton species abundance in Lake Kasumigaura (Japan) monitored monthly or biweekly since 1978. Ecological Research, 27, 837. 10.1007/s11284-012-0971-3

[gcb16485-bib-0091] Takamura, N. , Nakagawa, M. , & Hanazato, T. (2017). Zooplankton abundance in the pelagic region of Lake Kasumigaura (Japan): monthly data since 1980. Ecological Research, 32, 1.

[gcb16485-bib-0092] Thackeray, S. J. , De Ville, M. M. , Fletcher, J. M. , James, J. B. , Maberly, S. C. , Mackay, E. B. , & Winfield, I. J. (2015). Cumbrian Lakes plankton and fish data (1940 to 2013). NERC Environmental Information Data Centre (Dataset). 10.5285/1de49dab-c36e-4700-8b15-93a639ae4d55

[gcb16485-bib-0093] Thompson, P. L. , Isbell, F. , Loreau, M. , O'Connor, M. I. , & Gonzalez, A. (2021). The strength of the biodiversity–ecosystem function relationship depends on spatial scale. Proceedings of the Royal Society B: Biological Sciences, 285, 20180038. 10.1098/rspb.2018.0038 PMC6015867 29875295

[gcb16485-bib-0094] Tilman, D. , Isbell, F. , & Cowles, J. M. (2014). Biodiversity and ecosystem functioning. Annual Review of Ecology, Evolution, and Systematics, 45, 471–493.

[gcb16485-bib-0095] Uezu, A. , & Metzger, J. P. (2016). Time‐lag in responses of birds to atlantic forest fragmentation: Restoration opportunity and urgency. PLoS ONE, 11, e0147909. 26820548 10.1371/journal.pone.0147909 PMC4731062

[gcb16485-bib-0096] Vogt, R. J. , Beisner, B. E. , & Praire, Y. T. (2010). Functional diversity is positively associated with biomass for lake diatoms. Freshwater Biology, 55, 1636–1646.

[gcb16485-bib-0097] Walker, L. R. , Hölzel, N. , Marrs, R. , del Moral, R. , & Prach, K. (2014). Optimization of intervention levels in ecological restoration. Applied Vegetation Science, 17, 187–192.

[gcb16485-bib-0098] Walsh, J. R. , Lathrop, R. C. , & Vander Zanden, M. J. (2017). Invasive invertebrate predator, *Bythotrephes longimanus*, reverses trophic cascade in a north‐temperate lake. Limnology and Oceanography, 62, 2498–2509.

[gcb16485-bib-0099] Warren, P. H. , & Gaston, K. J. (1992). Predator–prey ratios: A special case of a general pattern? Philosophical Transactions of the Royal Society B: Biological Sciences, 338, 113–130.

[gcb16485-bib-0100] Watts, K. , Whytock, R. C. , Park, K. J. , Fuentes‐Montemayor, E. , Macgregor, N. A. , Duffield, S. , & McGowan, P. J. (2020). Ecological time lags and the journey towards conservation success. Nature Ecology and Evolution, 4, 304–311. 31988448 10.1038/s41559-019-1087-8

[gcb16485-bib-0101] Wernberg, T. , Smale, D. A. , Tuya, F. , Thomsen, M. S. , Langlois, T. J. , de Bettignies, T. , Bennett, S. , & Rousseaux, C. S. (2013). An extreme climatic event alters marine ecosystem structure in a global biodiversity hotspot. Nature Climate Change, 3, 78–82.

[gcb16485-bib-0102] Williams, N. F. , McRae, L. , Freeman, R. , Capdevila, P. , & Clements, C. F. (2021). Scaling the extinction vortex: Body size as a predictor of population dynamics close to extinction events. Ecology and Evolution, 11, 7069–7079. 10.1002/ece3.7555 34141276 PMC8207159

[gcb16485-bib-0103] Ye, H. , Deyle, E. R. , Gilarranz, L. J. , & Sugihara, G. (2015). Distinguishing time‐delayed causal interactions using convergent cross mapping. Scientific Reports, 5, 14750. 26435402 10.1038/srep14750 PMC4592974

[gcb16485-bib-0104] Ye, L. , Chang, C.‐W. , Matsuzaki, S. S. , Takamura, N. , Widdicombe, C. E. , & Hsieh, C. (2019). Functional diversity promotes phytoplankton resource use efficiency. Journal of Ecology, 107, 2353–2363.

[gcb16485-bib-0510] Zarnowitz, V. , & Ozyildirim, A. (2006). Time series decomposition and measurement of business cycles, trends and growth cycles. Journal of Monetary Economics, 53(7), 1717–1739. 10.1016/j.jmoneco.2005.03.015

[gcb16485-bib-0105] Zhu, J. , Jiang, L. , & Zhang, Y. (2016). Relationships between functional diversity and aboveground biomass production in the Northern Tibetan alpine grasslands. Scientific Reports, 6, 34105. 27666532 10.1038/srep34105 PMC5036173

[gcb16485-bib-0106] Zohary, T. (2004). Changes to the phytoplankton assemblage of Lake Kinneret after decades of a predictable, repetitive pattern. Freshwater Biology, 49, 1355–1371.

[gcb16485-bib-0511] Zohary, T. , Sukenik, A. , Berman‐Frank, I. , & Nishri, A. (2014). Lake Kinneret: Ecology and management. Springer Dordrecht. 10.1007/978-94-017-8944-8

