# Supplementary material for: Planktonic functional diversity changes in synchrony with lake ecosystem state
Source: Glob Chang Biol. 2022 Nov 12;29(3):686–701. doi: 10.1111/gcb.16485 (PMC10100413; doi:10.1111/gcb.16485)
Supplement: Supplementary file 1 — Data S1 [file GCB-29-686-s001.pdf]

Supplementary Material: Planktonic functional diversity changes in synchrony with lake ecosystem state

Duncan A. O'Brien [a*], duncan.a.obrien@gmail.com ORCID - 0000-0002-3420-5210

Gideon Gal [b], gal@ocean.org.il ORCID - 0000-0002-6962-4001

Stephen J. Thackeray [c], sjtr@ceh.ac.uk ORCID - 0000-0003-3274-2706

Shin-ichiro S. Matsuzaki [d], matsuzakiss@nies.go.jp ORCID - 0000-0003-2744-1343

Christopher F. Clements [a], c.clements@bristol.ac.uk ORCID - 0000-0001-5677-5401

[a] School of Biological Sciences, University of Bristol, Bristol, BS8 1TQ, UK

[b] Kinneret Limnological Laboratory, Israel Oceanographic & Limnological Research, PO Box 447, Migdal, Israel

[c] Lake Ecosystems Group, UK Centre for Ecology & Hydrology, Bailrigg, Lancaster, UK

[d] Biodiversity Division, National Institute for Environmental Studies, 16-2 Onogawa, Tsukuba, Ibaraki, 305-8506, Japan

[*]Corresponding author

Supplementary Methods

System state metrics

Five common measures of ecosystem state were used to provide a representation of the development of each lake's plankton community through time. The five metrics used were community composition, total plankton density, Fisher information (FI), the multivariate index of variability (MVI), and trophic ratio.

Community composition was represented by the first component of a principal component analysis using the monthly plankton species densities. Principal component analysis involves the reprojection of the species densities at each time point upon the eigenvectors of the dataset's covariance matrix. The first principal component is consequently the axis/eigenvector that maximises the variance of the projected density data and has previously been used in ecological systems to provide a dimensionally reduced variable representing the overall community structure (Hare & Mantua 2000; Andersen *et al.* 2009). Plankton densities were scaled to mean zero and unit variance to ensure each species had equal contribution to the coordinate axes.

Total plankton density is simply the sum of plankton densities at each time point (Kraemer *et al.* 2017) while trophic ratio is the division of total zooplankton density by total phytoplankton density (Jeppesen *et al.* 2011). Both of these metrics have been used previously to represent overall community functioning.

Fisher information (FI) is derived from information theory and attempts to quantify the amount of information that observable data can provide on an unmeasured parameter (Fisher

& Russell 1922). It consequently has been co-opted to act as an indicator of system dynamics and stability, where decreasing FI indicates decreasing system stability (Cabezas *et al.* 2010). Here, we used a simplified discrete time equation of Fisher and Russel (1922)'s mathematic proof following Karunanithi *et al.* (2008):

$$FI \approx 4 \sum_{i=1}^{m} [q_i - q_{i+1}]^2$$

where q_i^2 is the amplitude of the probability of observing states of the system at time window i and m is the number of possible 'states'. Possible states are defining by comparing the difference between temporally adjacent data windows to a reference 'uncertainty', typically suggested to be the standard deviation of each variable (in our case each plankton species' density) across the entire time series multiplied by 2 (Karunanithi et al. 2008). If the absolute difference in density is less than the reference deviation for all variables (i.e. species densities), then the windows are binned in to the same 'state'. In this study, we used a rolling window of five time points to ensure maximum coverage. While Fisher information has long been used in statistics, it has recently been suggested as an appropriate stability measure for ecosystems specifically identifying regime shifts (Ahmad et al. 2016; Konig et al. 2019) to warrant inclusion.

The multivariate index of variability was proposed by Brock & Carpenter (2006) as a measure of stability loss, where increasing MVI represents decreasing stability. The MVI is represented by the square root of the dominant eigenvalue of the covariance matrix of all plankton species across a rolling window of five time points. The use of the MVI in this study consequently gives an approximation of overall community variance, an important component of stability lacking from the four other state metrics used.

Fuzzily-coded trait dissimilarity

Fuzzy coding is an approach that has been successfully applied to multiple freshwater macroinvertebrate communities (Brown *et al.* 2018; Múrria *et al.* 2020), and has been suggested as an appropriate tool to circumvent some of the challenges non-species level data can bring to trait-based approaches in plankton (Martini *et al.* 2021). In practice, a fuzzy trait is sub-categorised and 'affinity' is the proportion of the taxon's species which fall within each of the fuzzily coded sub-categories (Gower 1971; de Bello *et al.* 2021). During this study, each of the lakes have sufficient monitoring documentation that genus level data identifies the species that have had their abundances pooled to form a taxon count. Therefore, we were able to code only species known to contribute to the count rather than coding all possible taxon members present in the trait databases. A fuzzily coded trait matrix was consequently uniquely constructed for each lake and plankton guild (phytoplankton vs zooplankton), and from it a dissimilarity matrix based on a modified Gower index was derived using the '*gawdis'* package in R (R Core Team 2020). This dissimilarity matrix was then Cailliez transformed (Cailliez 1983) to improve suitability for Euclidean-based analysis during construction of the 'trait space'. Our choice of a dissimilarity-based functional diversity methodology stems from our aim to quantify changes in trait space size through time (Mammola *et al.* 2021).

Functional diversity metrics

Functional diversity is usually estimated using three metrics (functional richness – FRic, functional evenness – FEve, and functional dispersion - FDis) which together attempt to quantify different aspects of the trait space available to a community at any one point in time (Laliberté & Legendre 2010). To achieve this, principal coordinate analysis is performed on the trait dissimilarity matrix to estimate species coordinates within the resulting

multidimensional space (Magneville *et al.* 2022). From these coordinates, the three functional diversity metrics can be calculated. Functional richness characterises the area encompassed by the community as measured by a convex hull, functional evenness is the length of the minimum spanning tree between species positions, and functional dispersion quantifies the average distance to the point of community 'gravity' (the centroid of community values weighted towards abundant species). Figure S2 gives a visual representation of these metrics.

Convergent cross mapping

Convergent cross mapping invokes Takens' embedding theorem (Takens 1981) which suggests that an underlying latent system/attractor manifold can be reconstructed from one or more related time series. Specifically, if an observed time series is considered a transformation of the manifold's states over time, transformed by an observation function consisting of stochasticity and observation error, then the manifold's reconstruction is possible by time-delay embedding the time series (Chang *et al.* 2017). As a result, if two time series, X and Y, share a manifold, a causal relationship between them can be assessed. Causality is identified by comparing the quality of prediction of one time series from the reconstructed system built upon the time-delay embedding of another (Runge *et al.* 2019); if X can be predicted using the reconstruction from Y, then X had a causal effect on Y. The reciprocal relationship is then also tested to establish whether bi-directional causation is present.

The appropriate embedding dimension for CCM was selected using nearest neighbour forecasting, specifically simplex projection (Sugihara & May 1990). Simplex projections generate forecasts of the manifold reconstruction and identifies the embedding dimension with the highest prediction skill (represented as the correlation between observed and

predicted values). In this analysis, we explored dimensions from 0 to 10 with an increment of 1, using $\tau = 1$ (a month) for embedding. Using the identified embedding dimension, CCM analysis was performed for each time-to-prediction lag/delay with a library size set to the maximum possible to improve prediction error and the likelihood of convergence (Sugihara *et al.* 2012).

References

Ahmad, N., Derrible, S., Eason, T. & Cabezas, H. (2016). Using Fisher information to track stability in multivariate systems. *Royal Society Open Science*, 3, 160582.

Andersen, T., Carstensen, J., Hernández-García, E. & Duarte, C.M. (2009). Ecological thresholds and regime shifts: approaches to identification. *Trends in Ecology & Evolution*, 24, 49–57.

de Bello, F., Botta-Dukát, Z., Lepš, J. & Fibich, P. (2021). Towards a more balanced combination of multiple traits when computing functional differences between species. *Methods in Ecology and Evolution*, 12, 443–448.

Brock, W. & Carpenter, S. (2006). Variance as a leading indicator of regime shift in ecosystem services. *Ecology and Society*, 11, 9.

Brown, L.E., Khamis, K., Wilkes, M., Blaen, P., Brittain, J.E., Carrivick, J.L., *et al.* (2018). Functional diversity and community assembly of river invertebrates show globally consistent responses to decreasing glacier cover. *Nature Ecology & Evolution*, 2, 325–333.

Cabezas, H., Campbell, D., Eason, T., Garmestani, A.S., Heberling, M.T., Hopton, M.E., *et al.* (2010). San Luis Basin sustainability metrics project: A methodology for evaluating regional sustainability. *Heberling, MT, Hopton, ME, Eds*, 119–136.

Cailliez, F. (1983). The analytical solution of the additive constant problem. *Psychometrika*, 48, 305–308.

Chang, C.-W., Ushio, M. & Hsieh, C. (2017). Empirical dynamic modeling for beginners. *Ecological Research*, 32, 785–796.

Fisher, R.A. & Russell, E.J. (1922). On the mathematical foundations of theoretical statistics. *Philosophical Transactions of the Royal Society of London. Series A, Containing Papers of a Mathematical or Physical Character*, 222, 309–368.

Gower, J.C. (1971). A general coefficient of similarity and some of its properties. *Biometrics*, 27, 857–871.

Hare, S.R. & Mantua, N.J. (2000). Empirical evidence for North Pacific regime shifts in 1977 and 1989. *Progress in Oceanography*, 47, 103–145.

Jeppesen, E., Nõges, P., Davidson, T.A., Haberman, J., Nõges, T., Blank, K., *et al.* (2011). Zooplankton as indicators in lakes: a scientific-based plea for including zooplankton in the ecological quality assessment of lakes according to the European Water Framework Directive (WFD). *Hydrobiologia*, 676, 279.

Karunanithi, A.T., Cabezas, H., Frieden, B.R. & Pawlowski, C.W. (2008). Detection and assessment of ecosystem regime shifts from Fisher Information. *Ecology and Society*, 13.

Konig, E., Cabezas, H. & Mayer, A.L. (2019). Detecting dynamic system regime boundaries with Fisher information: the case of ecosystems. *Clean Technologies and Environmental Policy*, 21, 1471–1483.
Kraemer, B.M., Mehner, T. & Adrian, R. (2017). Reconciling the opposing effects of warming on phytoplankton biomass in 188 large lakes. *Scientific Reports*, 7, 10762.
Laliberté, E. & Legendre, P. (2010). A distance-based framework for measuring functional diversity from multiple traits. *Ecology*, 91, 299–305.
Magneville, C., Loiseau, N., Albouy, C., Casajus, N., Claverie, T., Escalas, A., *et al.* (2022). mFD: an R package to compute and illustrate the multiple facets of functional diversity. *Ecography*, 2022.
Mammola, S., Carmona, C.P., Guillerme, T. & Cardoso, P. (2021). Concepts and applications in functional diversity. *Functional Ecology*, n/a.
Martini, S., Larras, F., Boyé, A., Faure, E., Aberle, N., Archambault, P., *et al.* (2021). Functional trait-based approaches as a common framework for aquatic ecologists. *Limnology and Oceanography*, 66, 965–994.
Múrria, C., Iturrarte, G. & Gutiérrez-Cánovas, C. (2020). A trait space at an overarching scale yields more conclusive macroecological patterns of functional diversity. *Global Ecology and Biogeography*, 29, 1729–1742.
R Core Team. (2020). R: A language and environment for statistical computing.
Runge, J., Bathiany, S., Bollt, E., Camps-Valls, G., Coumou, D., Deyle, E., *et al.* (2019). Inferring causation from time series in Earth system sciences. *Nature Communications*, 10, 2553.
Sugihara, G., May, R., Ye, H., Hsieh, C., Deyle, E., Fogarty, M., *et al.* (2012). Detecting causality in complex ecosystems. *Science (1979)*, 338, 496 LP – 500.
Sugihara, G. & May, R.M. (1990). Nonlinear forecasting as a way of distinguishing chaos from measurement error in time series. *Nature*, 344, 734–741.
Takens, F. (1981). Detecting strange attractors in turbulence. In: *Dynamical Systems and Turbulence, Warwick 1980* (eds. Rand, D. & Young, L.-S.). Springer Berlin Heidelberg, Berlin, Heidelberg, pp. 366–381.

Supplementary Figures

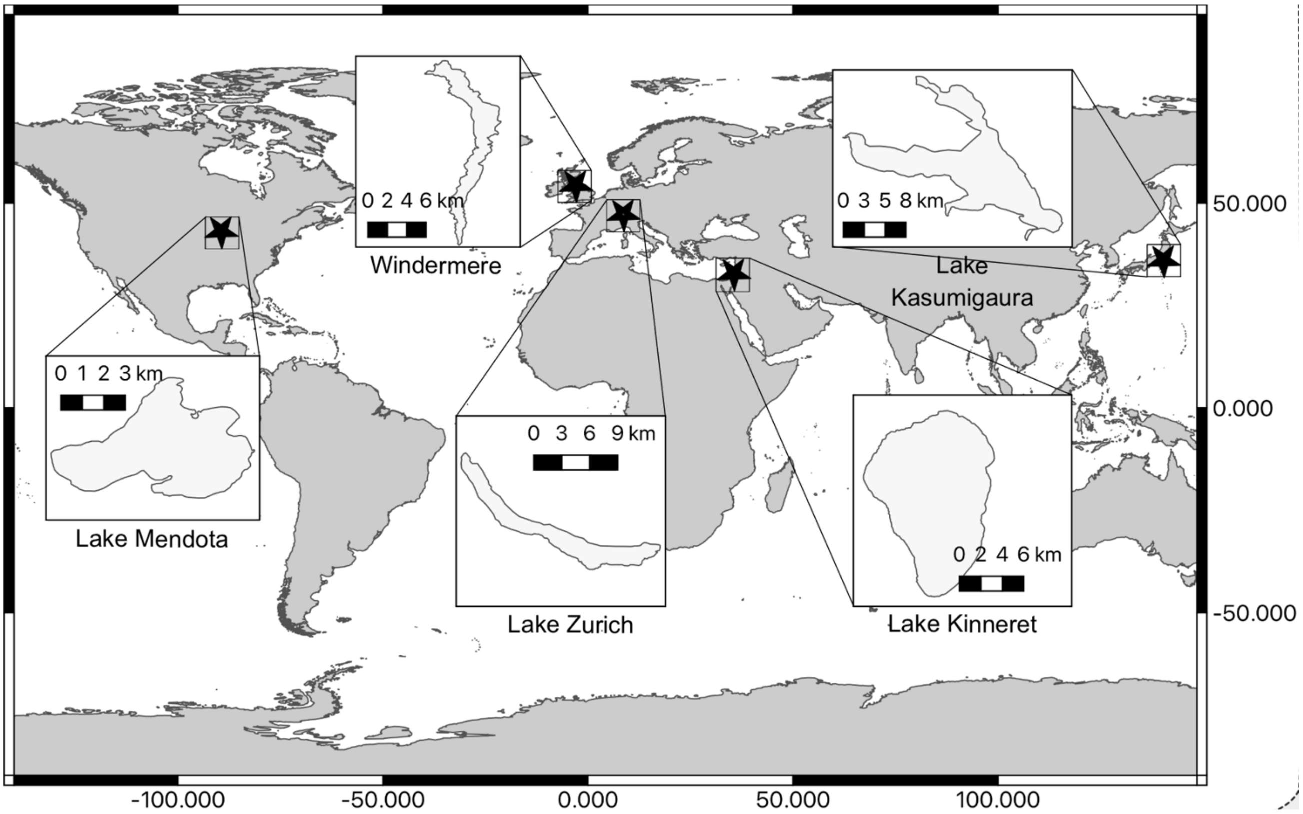

Figure S1. Locations of the five lake ecosystems used in this study and their relative surface areas. Map is projected in WGS 84.

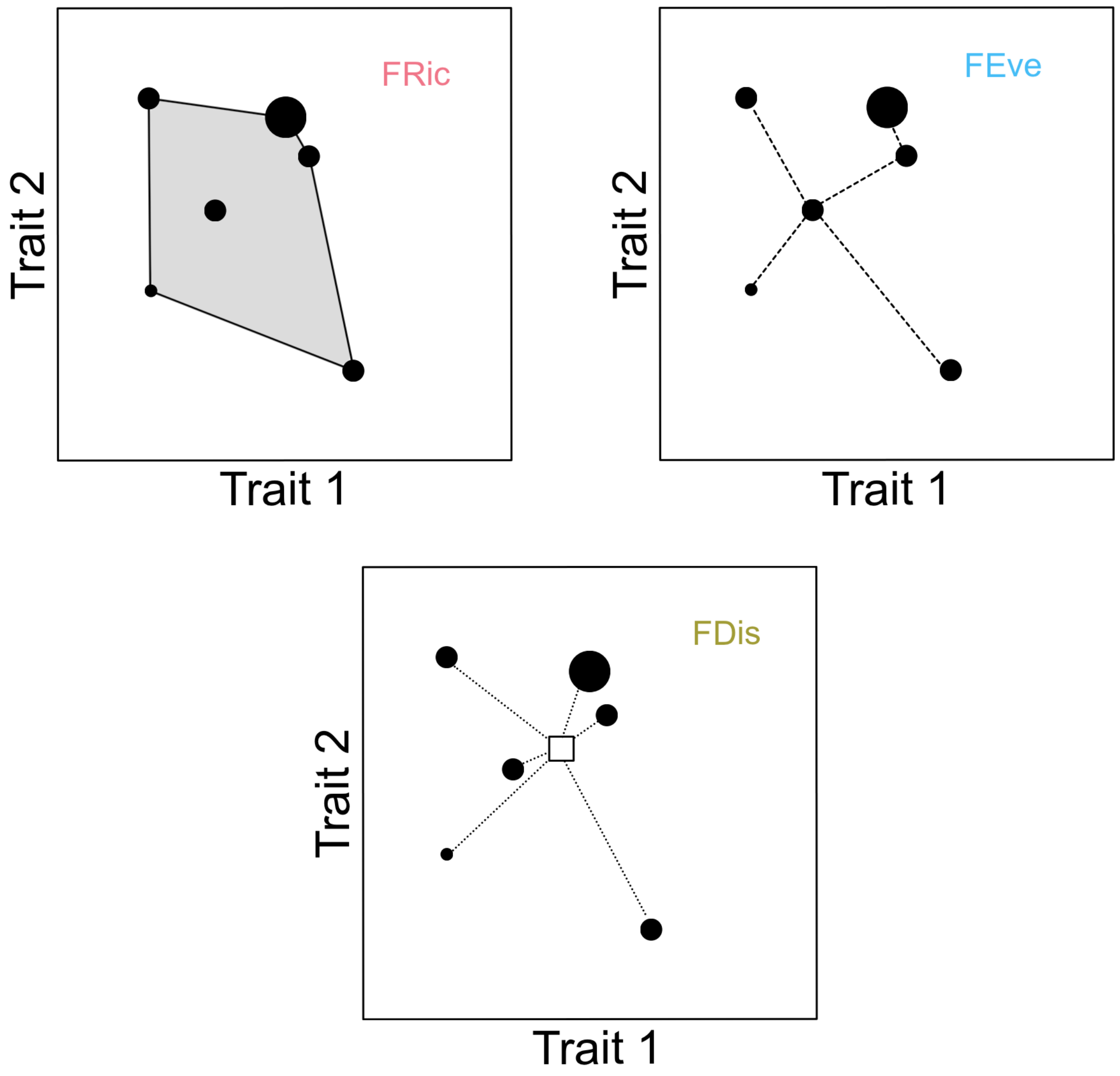

Figure S2. Diagrammatic representation of the three functional diversity metrics based upon trait dissimilarity. Points denote a species' trait value/coordinate within the trait space, with the size of the point proportional to the abundance of that species at time *t*. FRic is functional richness, FEve is functional evenness, and FDis is functional dispersion.

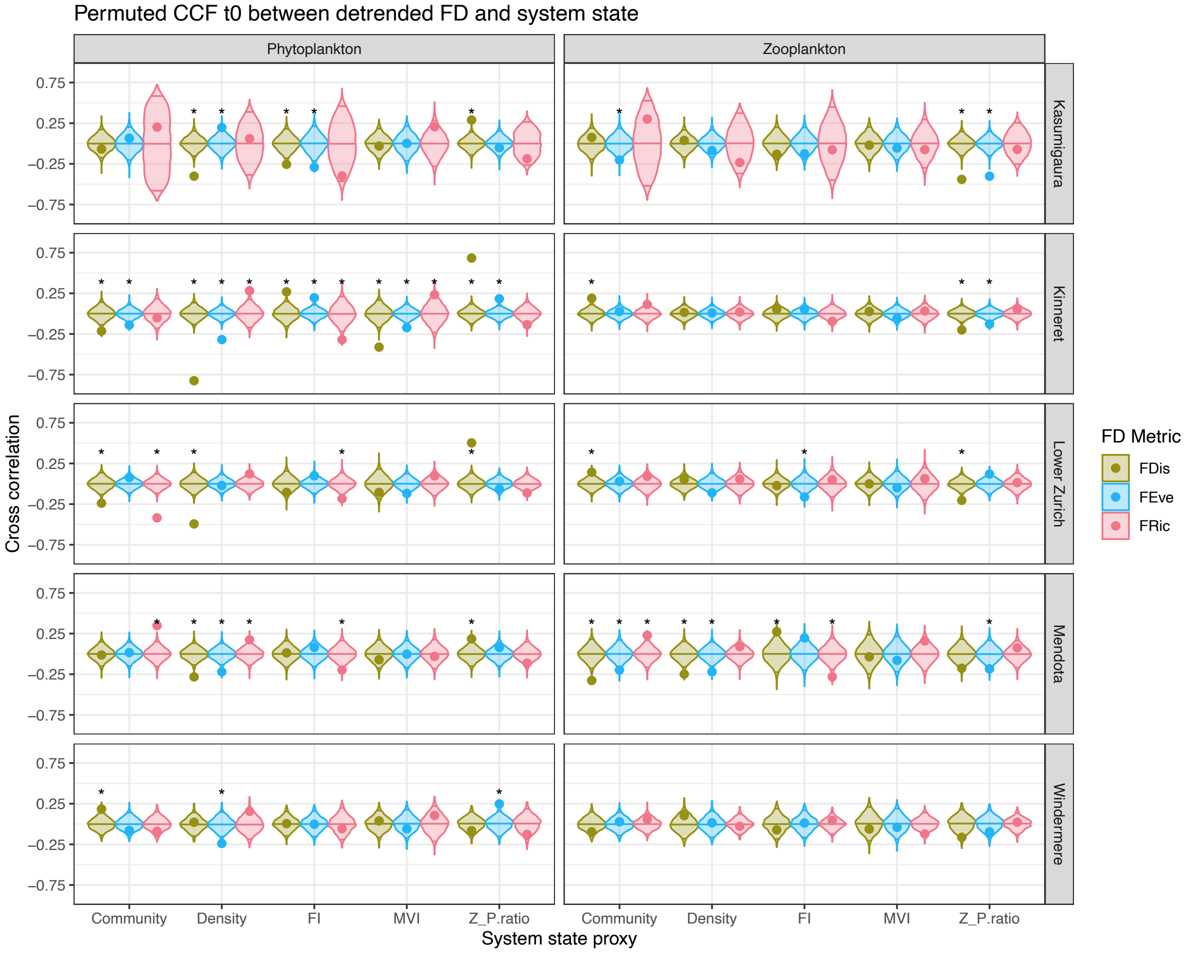

Figure S3. Density of the permuted instantaneous (Lag0) cross-correlation coefficients following 10,000 simulations between functional diversity and each of the system state metrics. Points represent the observed correlation coefficient and stars represent the ‘significant’ relationships (where the observed value transgresses the 2.5-97.5% confidence intervals).

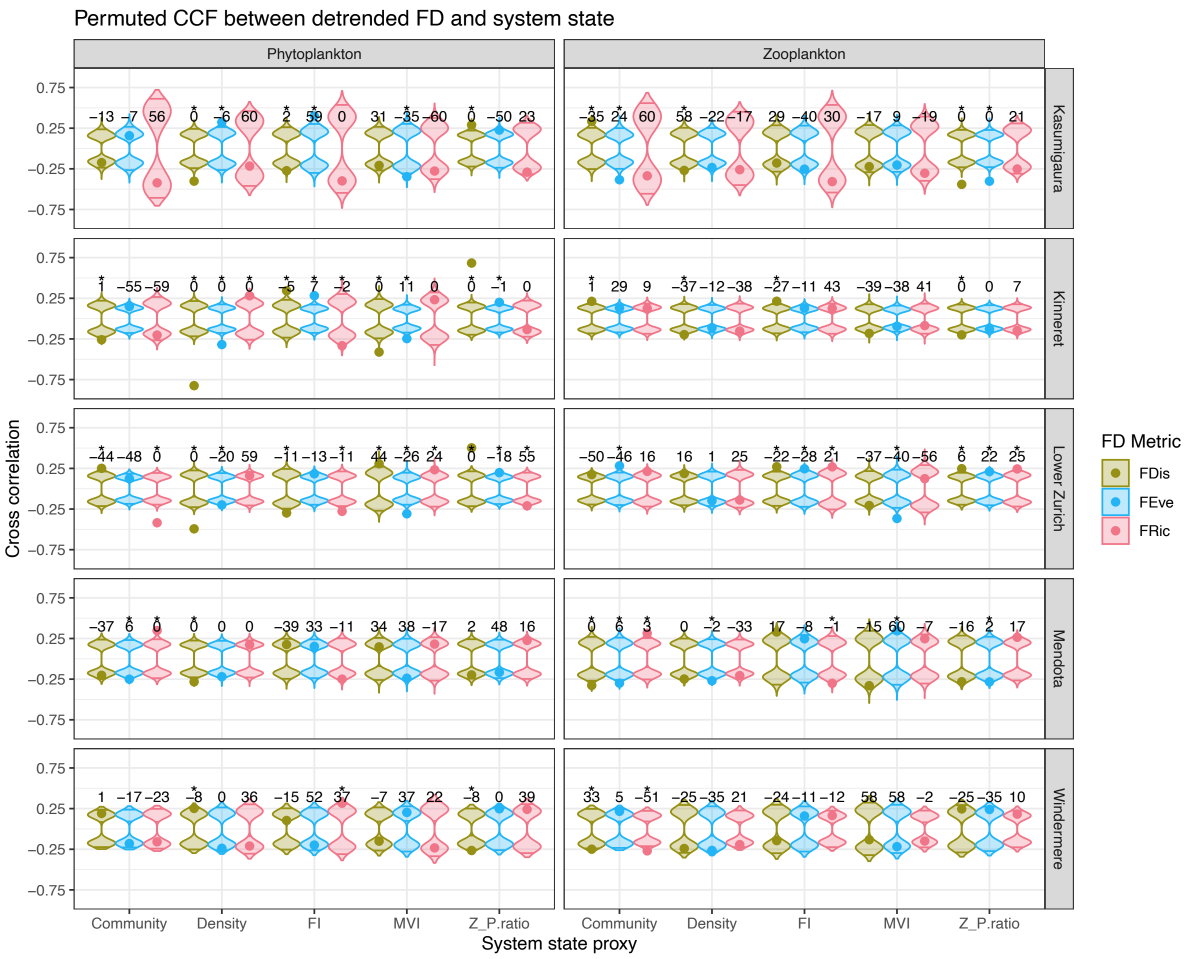

Figure S4. Density of permuted cross-correlation coefficients following 10,000 simulations between functional diversity and each of the system state metrics (LagX). Points represent the absolute strongest observed correlation coefficient and stars represent the ‘significant’ relationships (where the observed value transgresses the 2.5-97.5% confidence intervals). The reported number is the optimal lag (in months) that the observed value was identified. Positive lags indicate functional diversity lagging state, whereas negative lags indicate functional diversity leading state.

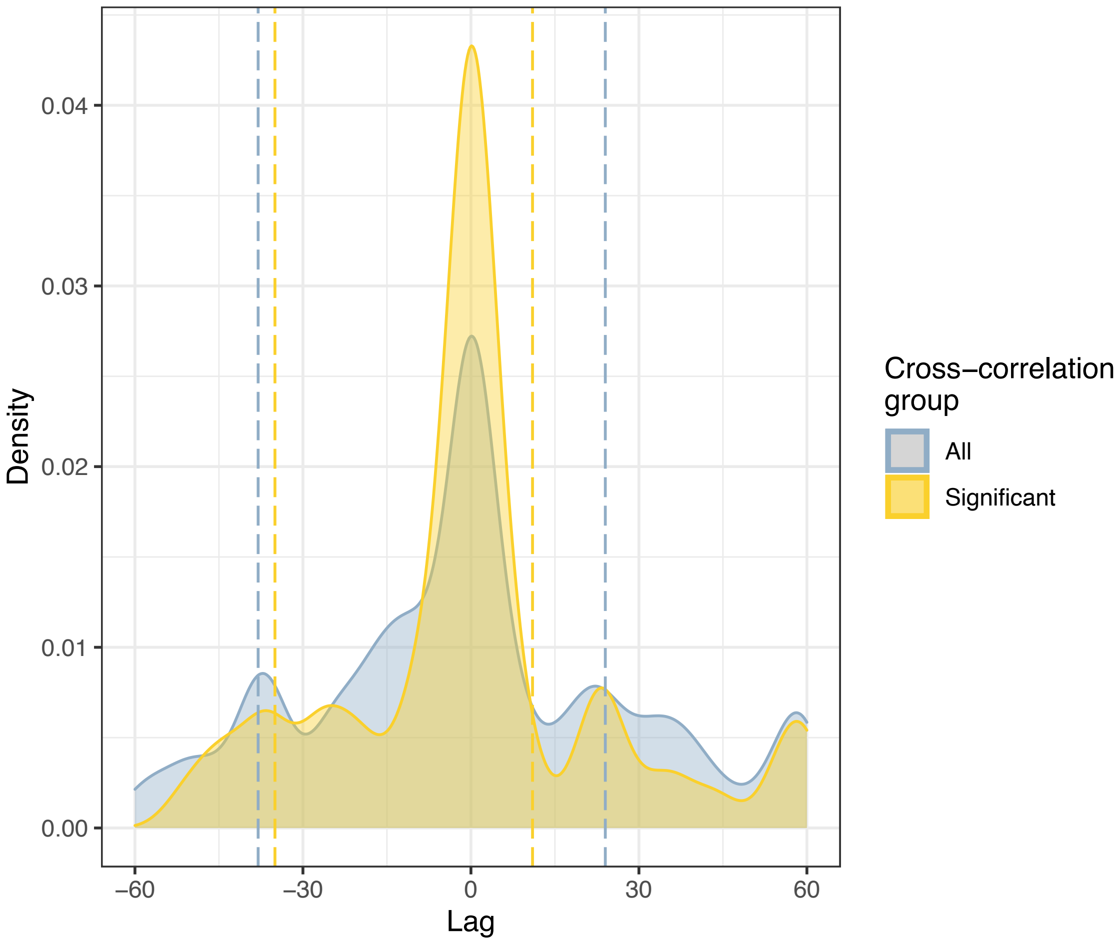

Figure S5. Density plots of the optimal lags identified by cross-correlations pooled across all functional diversity:system state combinations (blue area) or subset to significant combinations only (yellow area). Dashed, vertical lines indicate 10th and 90th quartiles.

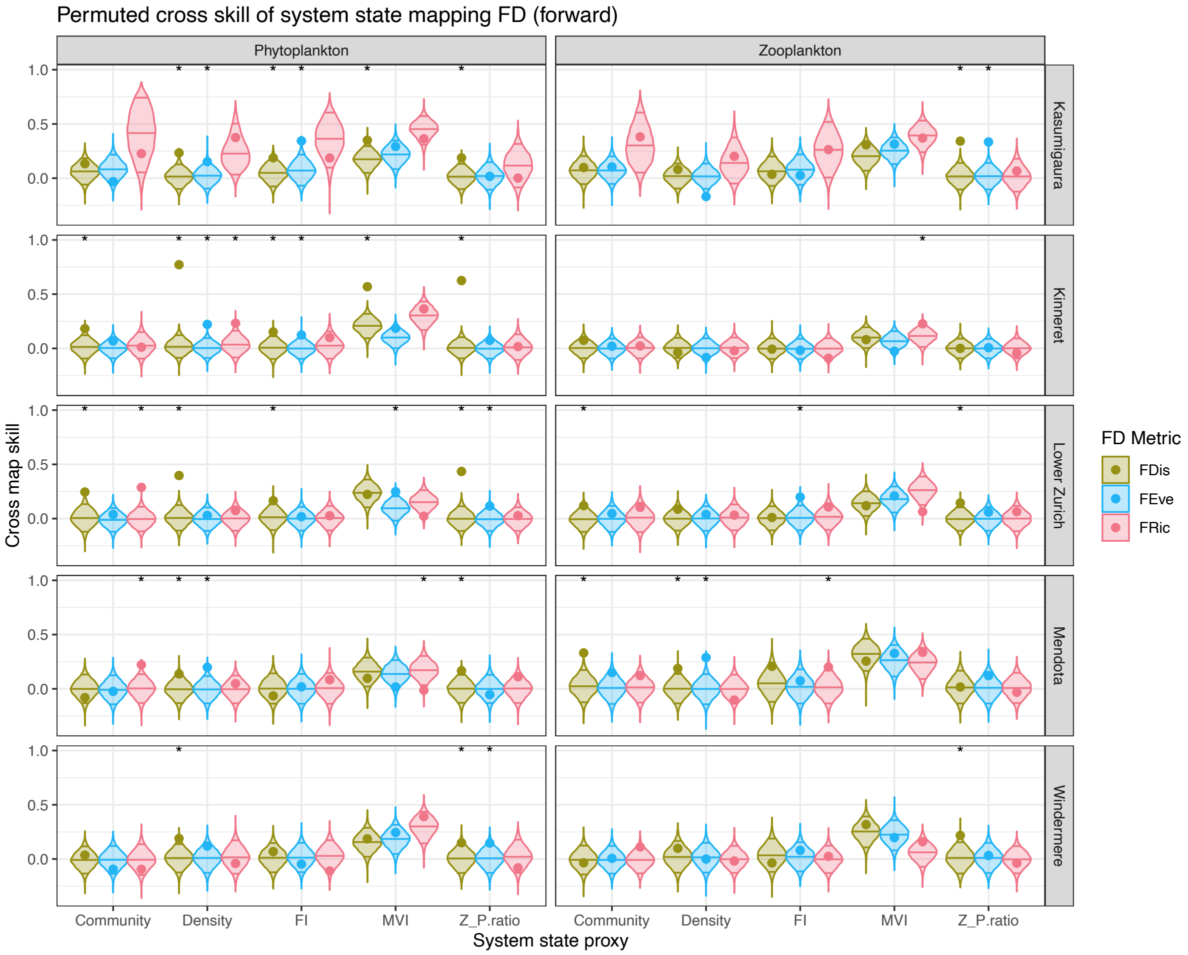

Figure S6. Density of instantaneous (Lag0) permuted cross mapping skills where the system state metrics map functional diversity following 1000 simulations. Points represent the observed cross map skill and stars represent the 'significant' relationships (where the observed value exceeds the 95% quantile). These points therefore represent the predicted strength of 'forward' causality where functional diversity 'causes' system state.

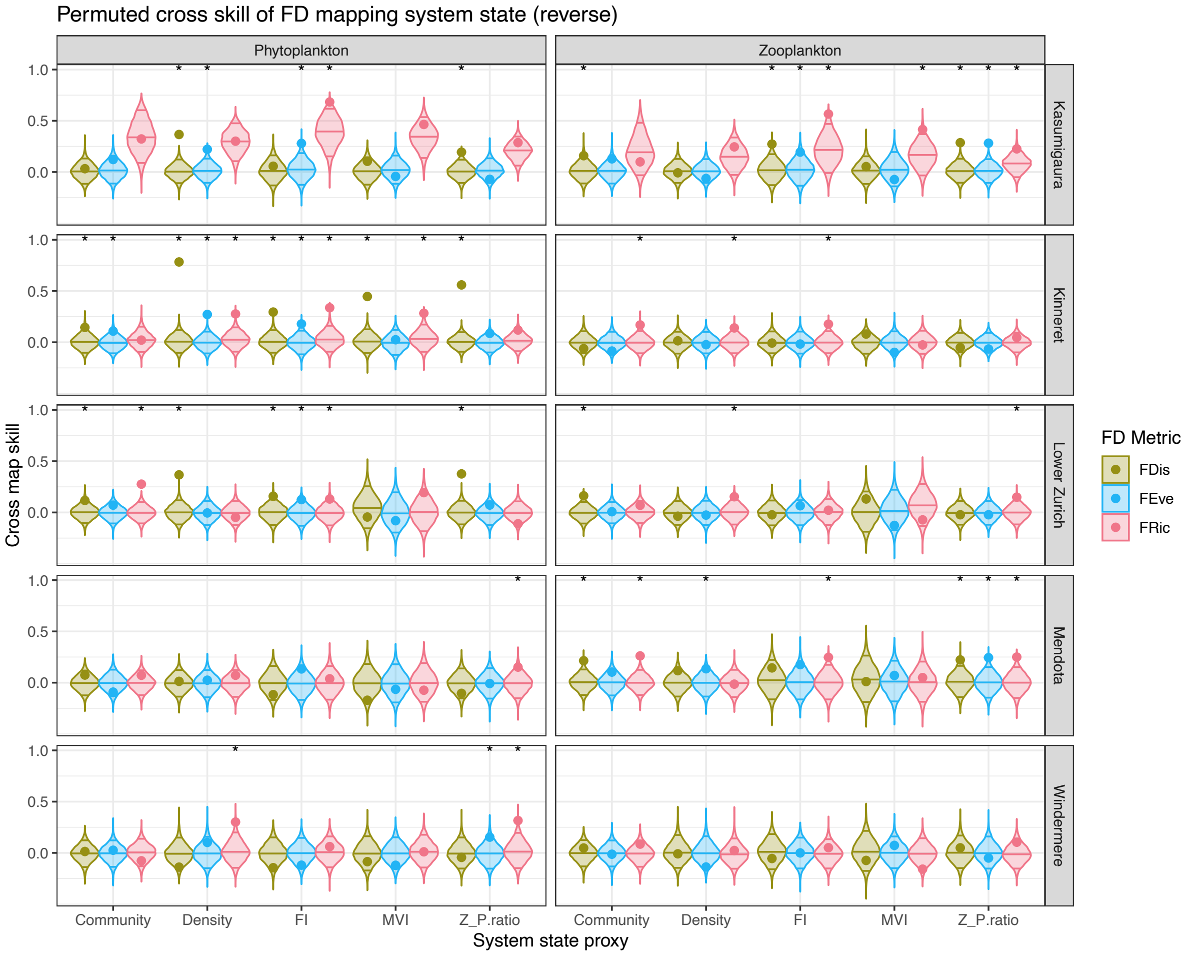

Figure S7. Density of instantaneous (Lag0) permuted cross mapping skills where functional diversity maps system state following 1000 simulations. Points represent the observed cross map skill and stars represent the 'significant' relationships (where the observed value exceeds the 95% quantile). These points therefore represent the predicted strength of 'reverse' causality where system state 'causes' functional diversity.

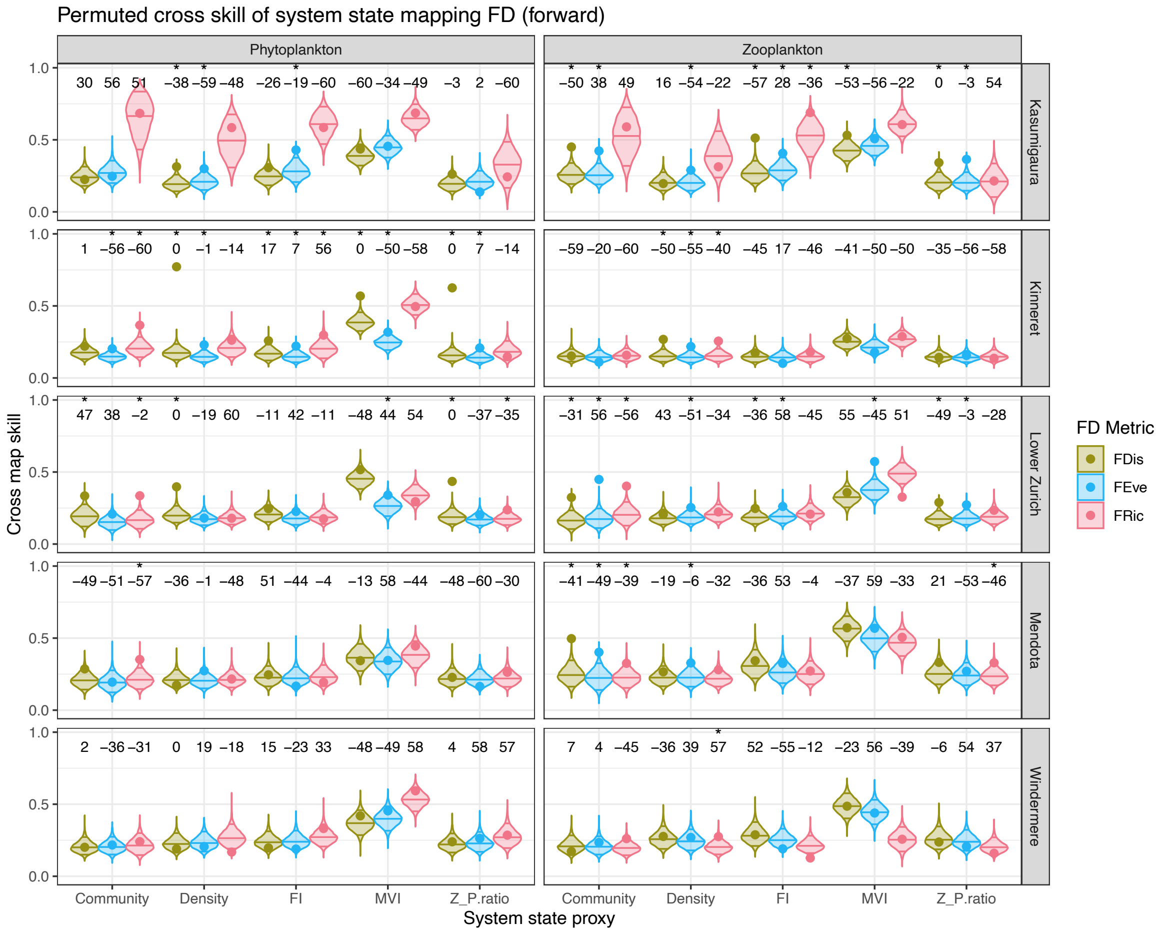

Figure S8. Density of permuted cross mapping skills (LagX) where the system state metrics map functional diversity following 10,000 simulations. Points represent the observed cross map skill and stars represent the 'significant' relationships (where the observed value exceeds the 95% quantile). These points therefore represent the predicted strength of 'forward' causality where functional diversity 'causes' system state. Optimal lags (in months) are indicated by the numbers above each distribution. Negative lags indicate functional diversity leading state whereas positive lags indicate generalised synchrony.

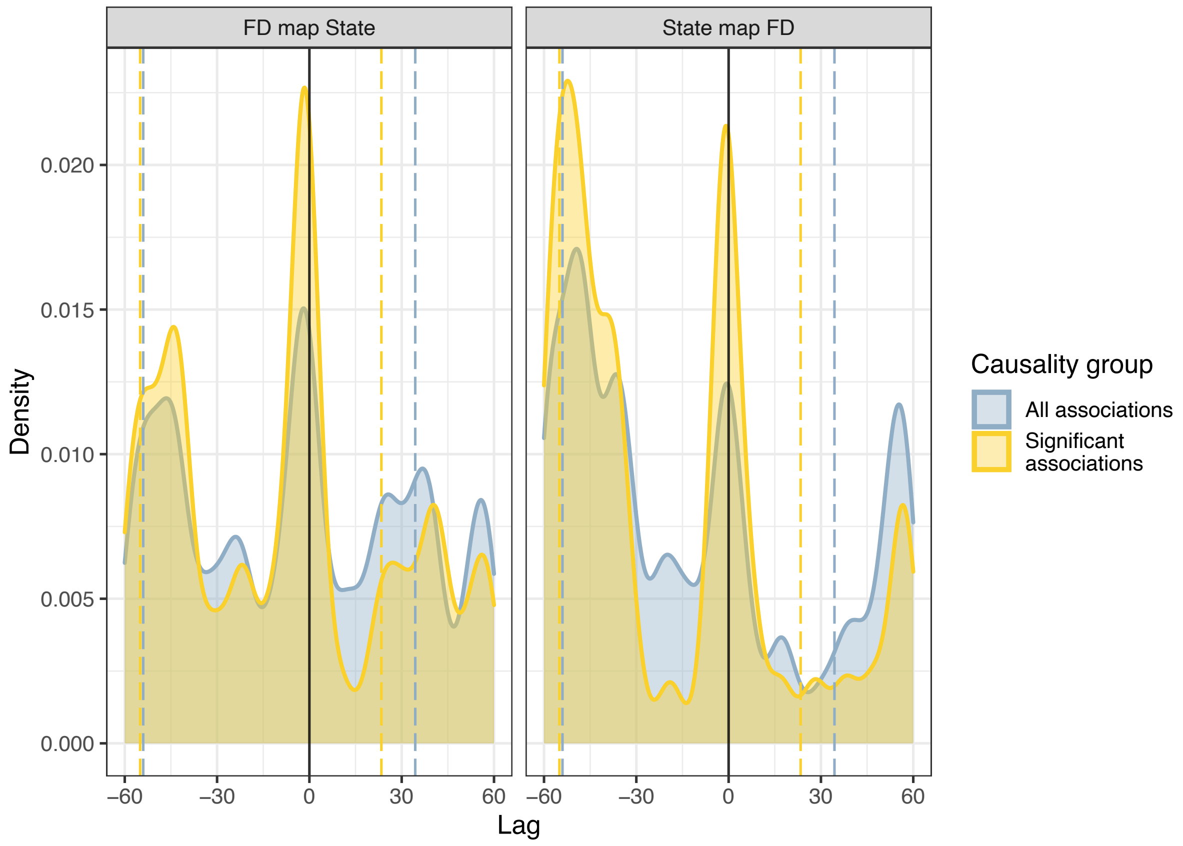

Figure S9. Density plots of the optimal lags identified by forward (State map FD) and reverse (FD map State) cross mappings pooled across all functional diversity:system state combinations (blue area) or subset to significant combinations only (yellow area). Dashed, vertical lines indicate 10th and 90th quartiles.

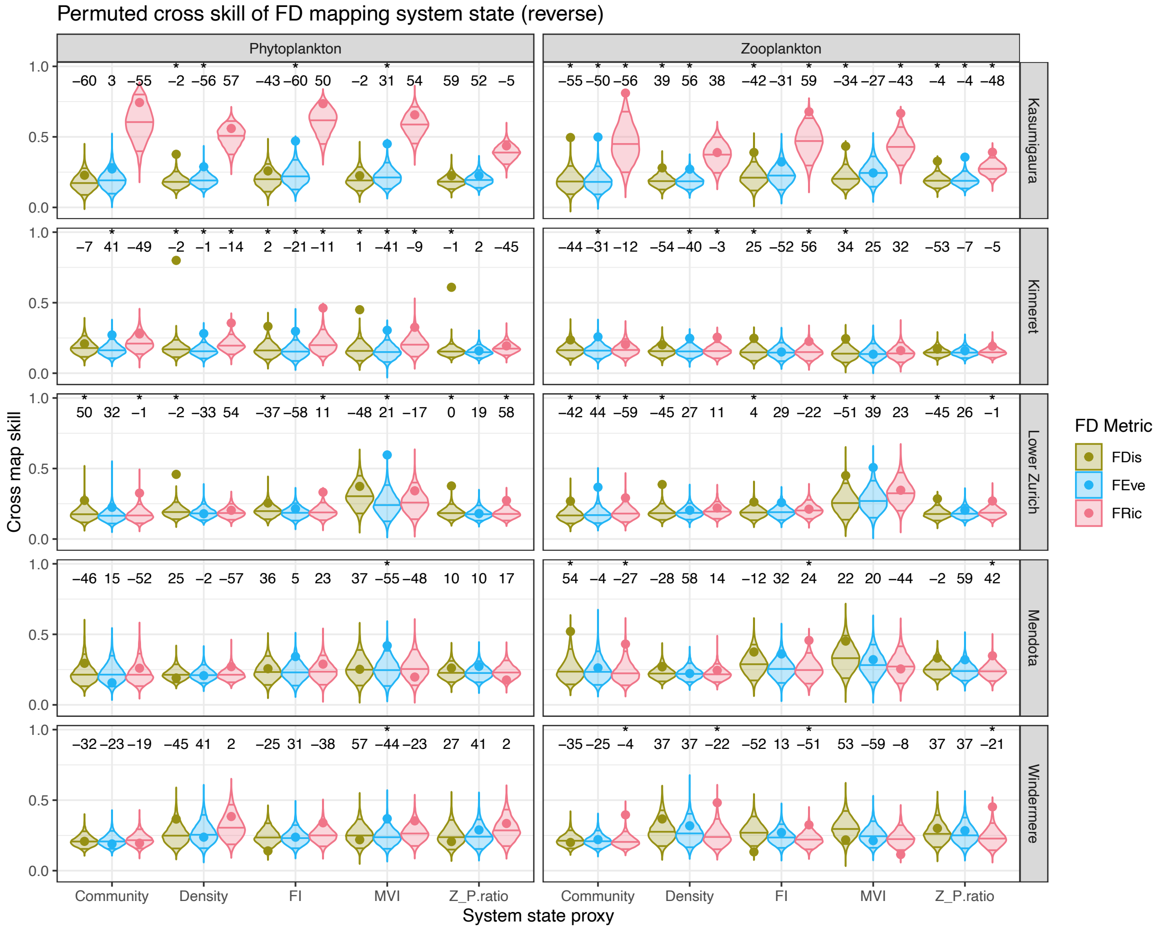

Figure S10. Density of permuted cross mapping skills (LagX) where functional diversity maps system state following 10,000 simulations. Points represent the observed cross map skill and stars represent the 'significant' relationships (where the observed value exceeds the 95% quantile). These points therefore represent the predicted strength of 'reverse' causality where system state 'causes' functional diversity. Optimal lags (in months) are indicated by the numbers above each distribution. Negative lags indicate functional diversity leading state whereas positive lags indicate generalised synchrony.

Supplementary Tables

Table S1. Summary statistics for each functional diversity-system state correlation at Lag0. Variation is reported as standard errors.

**Trophic Guild**	**Functional diversity metric**	**System state metric**	**Median correlation**	**Number of significant lakes**	**Proportion significant**
Phytoplankton	FDis	Community	-0.068 ± 0.034	3	0.6
		Density	-0.402 ± 0.062	4	0.8
		FI	0.005 ± 0.038	2	0.4
		MVI	-0.072 ± 0.035	1	0.2
		Z_P.ratio	0.29 ± 0.059	4	0.8
	FEve	Community	0.016 ± 0.019	1	0.2
		Density	-0.22 ± 0.042	4	0.8
		FI	0.081 ± 0.037	2	0.4
		MVI	-0.059 ± 0.015	1	0.2
		Z_P.ratio	0.079 ± 0.028	2	0.4
	FRic	Community	-0.051 ± 0.059	2	0.4
		Density	0.157 ± 0.016	2	0.4
		FI	-0.197 ± 0.026	3	0.6
		MVI	0.106 ± 0.021	1	0.2
		Z_P.ratio	-0.127 ± 0.006	0	0
Zooplankton	FDis	Community	0.075 ± 0.042	3	0.6
		Density	0.039 ± 0.028	1	0.2
		FI	-0.023 ± 0.032	1	0.2
		MVI	-0.02 ± 0.007	0	0
		Z_P.ratio	-0.2 ± 0.023	3	0.6
	FEve	Community	0.026 ± 0.025	2	0.4
		Density	-0.087 ± 0.019	1	0.2
		FI	0.012 ± 0.029	1	0.2
		MVI	-0.056 ± 0.003	0	0
		Z_P.ratio	-0.128 ± 0.037	3	0.6
	FRic	Community	0.114 ± 0.02	1	0.2
		Density	0.02 ± 0.026	0	0
		FI	-0.077 ± 0.027	1	0.2
		MVI	0.032 ± 0.022	0	0
		Z_P.ratio	0.023 ± 0.011	0	0

Table S2. Summary statistics for each functional diversity-system state cross correlation across lags (LagX). Variation is reported as standard errors.

Trophic Guild	Functional diversity metric	System state metric	Median correlation	Median lag	Number of significant lakes	Proportion significant
Phytoplankton	FDis	Community	-0.173 ± 0.048	-13 ± 4.22	2	0.4
		Density	-0.402 ± 0.078	0 ± 0.72	5	1
		FI	0.104 ± 0.057	-11 ± 3.12	3	0.6
		MVI	-0.15 ± 0.058	31 ± 4.5	2	0.4
		Z_P.ratio	0.29 ± 0.084	0 ± 0.78	4	0.8
	FEve	Community	0.124 ± 0.04	-17 ± 5.27	1	0.2
		Density	-0.22 ± 0.052	0 ± 1.73	3	0.6
		FI	0.183 ± 0.045	33 ± 6.07	2	0.4
		MVI	-0.246 ± 0.045	11 ± 6.86	3	0.6
		Z_P.ratio	0.2 ± 0.035	-1 ± 7.1	2	0.4
	FRic	Community	-0.203 ± 0.063	0 ± 8.37	2	0.4
		Density	0.162 ± 0.047	36 ± 5.98	1	0.2
		FI	-0.278 ± 0.058	-2 ± 3.98	3	0.6
		MVI	0.181 ± 0.052	0 ± 6.9	1	0.2
		Z_P.ratio	-0.135 ± 0.05	23 ± 4.23	1	0.2
Zooplankton	FDis	Community	0.174 ± 0.059	0 ± 6.55	4	0.8
		Density	-0.241 ± 0.038	0 ± 7.47	2	0.4
		FI	0.213 ± 0.048	-22 ± 5.27	2	0.4
		MVI	-0.206 ± 0.015	-17 ± 7.92	0	0
		Z_P.ratio	-0.2 ± 0.063	0 ± 2.59	3	0.6
	FEve	Community	0.131 ± 0.062	6 ± 5.94	3	0.6
		Density	-0.234 ± 0.014	-12 ± 2.96	1	0.2
		FI	0.159 ± 0.041	-11 ± 2.77	1	0.2
		MVI	-0.203 ± 0.054	9 ± 9.8	2	0.4
		Z_P.ratio	-0.128 ± 0.058	0 ± 4.11	3	0.6
	FRic	Community	0.125 ± 0.059	9 ± 7.92	2	0.4
		Density	-0.192 ± 0.01	-17 ± 5.95	0	0
		FI	0.126 ± 0.061	21 ± 4.5	2	0.4
		MVI	-0.086 ± 0.044	-7 ± 6.97	0	0
		Z_P.ratio	0.182 ± 0.048	17 ± 1.5	1	0.2

Table S3. Summary statistics for each functional diversity-system state cross mapping at Lag0. This is the forward relationship (system state maps functional diversity) where a significant relationship suggests diversity causes state. Variation is reported as standard errors.

Trophic Guild	Functional diversity metric	System state metric	Median correlation	Number of significant lakes	Proportion significant
Phytoplankton	FDis	Community	0.134 ± 0.03	2	0.4
		Density	0.233 ± 0.05	5	1
		FI	0.152 ± 0.02	3	0.6
		MVI	0.223 ± 0.04	2	0.4
		Z_P.ratio	0.188 ± 0.04	5	1
	FEve	Community	-0.023 ± 0.01	0	0
		Density	0.152 ± 0.02	3	0.6
		FI	0.018 ± 0.03	2	0.4
		MVI	0.244 ± 0.02	1	0.2
		Z_P.ratio	0.075 ± 0.02	2	0.4
	FRic	Community	0.222 ± 0.03	2	0.4
		Density	0.074 ± 0.03	1	0.2
		FI	0.084 ± 0.02	0	0
		MVI	0.364 ± 0.04	1	0.2
		Z_P.ratio	0.017 ± 0.01	0	0
Zooplankton	FDis	Community	0.098 ± 0.03	2	0.4
		Density	0.087 ± 0.02	1	0.2
		FI	0.01 ± 0.02	0	0
		MVI	0.255 ± 0.02	0	0
		Z_P.ratio	0.144 ± 0.03	3	0.6
	FEve	Community	0.046 ± 0.01	0	0
		Density	0 ± 0.04	1	0.2
		FI	0.074 ± 0.02	1	0.2
		MVI	0.209 ± 0.03	0	0
		Z_P.ratio	0.06 ± 0.03	1	0.2
	FRic	Community	0.111 ± 0.03	0	0
		Density	-0.019 ± 0.02	0	0
		FI	0.107 ± 0.03	1	0.2
		MVI	0.227 ± 0.03	1	0.2
		Z_P.ratio	-0.032 ± 0.01	0	0

Table S4. Summary statistics for each functional diversity-system state cross mapping at Lag0. This is the reverse relationship (functional diversity maps system state) where a significant relationship suggests diversity is caused by state. Variation is reported as standard errors.

Trophic Guild	Functional diversity metric	System state metric	Median correlation	Number of significant lakes	Proportion significant
Phytoplankton	FDis	Community	0.074 ± 0.01	2	0.4
		Density	0.366 ± 0.07	3	0.6
		FI	0.057 ± 0.04	2	0.4
		MVI	-0.045 ± 0.05	1	0.2
		Z_P.ratio	0.194 ± 0.06	3	0.6
	FEve	Community	0.071 ± 0.02	1	0.2
		Density	0.102 ± 0.02	2	0.4
		FI	0.134 ± 0.03	3	0.6
		MVI	-0.065 ± 0.01	0	0
		Z_P.ratio	0.072 ± 0.02	1	0.2
	FRic	Community	0.072 ± 0.03	1	0.2
		Density	0.276 ± 0.03	2	0.4
		FI	0.131 ± 0.05	3	0.6
		MVI	0.194 ± 0.04	1	0.2
		Z_P.ratio	0.152 ± 0.03	2	0.4
Zooplankton	FDis	Community	0.16 ± 0.02	3	0.6
		Density	-0.007 ± 0.01	0	0
		FI	-0.008 ± 0.03	1	0.2
		MVI	0.053 ± 0.02	0	0
		Z_P.ratio	0.05 ± 0.03	2	0.4
	FEve	Community	0.007 ± 0.02	0	0
		Density	-0.026 ± 0.02	1	0.2
		FI	0.066 ± 0.02	1	0.2
		MVI	-0.073 ± 0.02	0	0
		Z_P.ratio	-0.022 ± 0.03	2	0.4
	FRic	Community	0.099 ± 0.02	2	0.4
		Density	0.14 ± 0.02	2	0.4
		FI	0.177 ± 0.04	3	0.6
		MVI	-0.026 ± 0.04	1	0.2
		Z_P.ratio	0.15 ± 0.02	3	0.6

Table S5. Causality comparison for lag0/instantaneous cross mappings between functional diversity and system state. The proportion of lakes with forward, reverse, bi-directional and zero causality are reported. Variation is reported as standard errors.

Trophic Guild	Functional diversity metric	System state metric	Proportion of lakes forward causal	Proportion of lakes reverse causal	Proportion of lakes bi-directionally causal	Proportion of lakes non-causal
Phytoplankton	FDis	Community	0	0	0.4	0.6
		Density	0.4	0	0.6	0
		FI	0.2	0	0.4	0.4
		MVI	0.2	0	0.2	0.6
		Z_P.ratio	0.4	0	0.6	0
	FEve	Community	0	0.2	0	0.8
		Density	0.2	0	0.4	0.4
		FI	0	0.2	0.4	0.4
		MVI	0.2	0	0	0.8
		Z_P.ratio	0.2	0	0.2	0.6
	FRic	Community	0.2	0	0.2	0.6
		Density	0	0.2	0.2	0.6
		FI	0	0.6	0	0.4
		MVI	0.2	0.2	0	0.6
		Z_P.ratio	0	0.4	0	0.6
Zooplankton	FDis	Community	0	0.2	0.4	0.4
		Density	0.2	0	0	0.8
		FI	0	0.2	0	0.8
		MVI	0	0	0	1
		Z_P.ratio	0.4	0.2	0.2	0.2
	FEve	Community	0	0	0	1
		Density	0	0	0.2	0.8
		FI	0.2	0.2	0	0.6
		MVI	0	0	0	1
		Z_P.ratio	0	0.2	0.2	0.6
	FRic	Community	0	0.4	0	0.6
		Density	0	0.4	0	0.6
		FI	0	0.4	0.2	0.4
		MVI	0.2	0.2	0	0.6
		Z_P.ratio	0	0.6	0	0.4

Table S6. Summary statistics for each functional diversity-system state cross mapping across lags (LagX). This is the forward relationship (system state maps functional diversity) where a significant relationship suggests diversity causes state. Variation is reported as standard errors.

Trophic Guild	Functional diversity metric	System state metric	Median correlation	Median lag	Number of significant lakes	Proportion significant
Phytoplankton	FDis	Community	0.224 ± 0.01	2 ± 7.30	1	0.2
		Density	0.314 ± 0.05	0 ± 4.06	3	0.6
		FI	0.245 ± 0.01	15 ± 5.91	1	0.2
		MVI	0.436 ± 0.02	-48 ± 5.16	1	0.2
		Z_P.ratio	0.262 ± 0.03	0 ± 4.34	2	0.4
	FEve	Community	0.209 ± 0.00	-36 ± 10.55	1	0.2
		Density	0.23 ± 0.01	-1 ± 5.88	2	0.4
		FI	0.221 ± 0.02	-19 ± 6.61	2	0.4
		MVI	0.345 ± 0.01	-34 ± 10.57	2	0.4
		Z_P.ratio	0.202 ± 0.01	2 ± 9.06	1	0.2
	FRic	Community	0.352 ± 0.03	-31 ± 9.20	3	0.6
		Density	0.216 ± 0.04	-18 ± 8.83	0	0
		FI	0.297 ± 0.03	-4 ± 8.90	1	0.2
		MVI	0.496 ± 0.03	-44 ± 11.70	0	0
		Z_P.ratio	0.243 ± 0.01	-30 ± 8.85	1	0.2
Zooplankton	FDis	Community	0.324 ± 0.03	-41 ± 5.12	3	0.6
		Density	0.266 ± 0.01	-19 ± 7.64	1	0.2
		FI	0.288 ± 0.03	-36 ± 8.71	2	0.4
		MVI	0.486 ± 0.03	-37 ± 8.63	1	0.2
		Z_P.ratio	0.289 ± 0.02	-6 ± 5.61	2	0.4
	FEve	Community	0.402 ± 0.03	4 ± 8.50	3	0.6
		Density	0.269 ± 0.01	-51 ± 8.29	4	0.8
		FI	0.261 ± 0.02	28 ± 9.07	2	0.4
		MVI	0.507 ± 0.03	-45 ± 11.84	1	0.2
		Z_P.ratio	0.27 ± 0.02	-3 ± 9.02	2	0.4
	FRic	Community	0.325 ± 0.03	-45 ± 9.01	2	0.4
		Density	0.275 ± 0.01	-32 ± 8.07	2	0.4
		FI	0.208 ± 0.05	-36 ± 3.88	1	0.2
		MVI	0.326 ± 0.03	-33 ± 8.04	0	0
		Z_P.ratio	0.215 ± 0.02	-28 ± 10.11	1	0.2

Table S7. Summary statistics for each functional diversity-system state cross mapping across lags (LagX). This is the reverse relationship (functional diversity maps system state) where a significant relationship suggests diversity is caused by state. Variation is reported as standard errors.

Trophic Guild	Functional diversity metric	System state metric	Median correlation	Median lag	Number of significant lakes	Proportion significant
Phytoplankton	FDis	Community	0.228 ± 0.008	-32 ± 8.65	1	0.2
		Density	0.376 ± 0.045	-2 ± 5.03	3	0.6
		FI	0.256 ± 0.014	-25 ± 6.52	1	0.2
		MVI	0.252 ± 0.021	1 ± 8.08	1	0.2
		Z_P.ratio	0.261 ± 0.033	10 ± 5.01	2	0.4
	FEve	Community	0.225 ± 0.01	15 ± 5.04	1	0.2
		Density	0.238 ± 0.009	-2 ± 7.34	2	0.4
		FI	0.297 ± 0.02	-21 ± 7.92	2	0.4
		MVI	0.42 ± 0.022	-41 ± 8.06	5	1
		Z_P.ratio	0.226 ± 0.011	19 ± 4.21	0	0
	FRic	Community	0.279 ± 0.044	-49 ± 4.79	1	0.2
		Density	0.356 ± 0.027	2 ± 9.62	1	0.2
		FI	0.339 ± 0.036	11 ± 6.69	2	0.4
		MVI	0.342 ± 0.034	-17 ± 7.58	1	0.2
		Z_P.ratio	0.274 ± 0.021	2 ± 7.46	1	0.2
Zooplankton	FDis	Community	0.268 ± 0.03	-42 ± 8.88	3	0.6
		Density	0.28 ± 0.015	-28 ± 9	2	0.4
		FI	0.263 ± 0.021	-12 ± 6.38	3	0.6
		MVI	0.433 ± 0.023	22 ± 8.99	3	0.6
		Z_P.ratio	0.3 ± 0.013	-4 ± 7.3	2	0.4
	FEve	Community	0.262 ± 0.023	-25 ± 7.19	3	0.6
		Density	0.248 ± 0.009	37 ± 7.99	2	0.4
		FI	0.27 ± 0.016	13 ± 7.54	1	0.2
		MVI	0.244 ± 0.028	20 ± 8.22	1	0.2
		Z_P.ratio	0.284 ± 0.016	26 ± 5.59	1	0.2
	FRic	Community	0.396 ± 0.046	-27 ± 5.01	4	0.8
		Density	0.255 ± 0.023	11 ± 4.43	2	0.4
		FI	0.325 ± 0.039	24 ± 9.7	3	0.6
		MVI	0.255 ± 0.044	-8 ± 7.13	1	0.2
		Z_P.ratio	0.348 ± 0.02	-5 ± 6.57	4	0.8

Table S8. Causality comparison for cross mappings between functional diversity and system state, across lags. The proportion of lakes with forward, reverse, bi-directional and zero causality are reported. Variation is reported as standard errors.

**Trophic Guild**	**Functional diversity metric**	**System state metric**	**Proportion of lakes forward causal**	**Proportion of lakes reverse causal**	**Proportion of lakes bi-directionally causal**	**Proportion of lakes non-causal**	**Median difference in lag (lagReverse – lagForward)**
Phytoplankton	FDis	Community	0	0	0.2	0.8	-25.2
		Density	0	0	0.6	0.4	9.6
		FI	0	0	0.2	0.8	-22.6
		MVI	0	0	0.2	0.8	42.8
		Z_P.ratio	0	0	0.4	0.6	28.4
	FEve	Community	0	0	0.2	0.8	23.4
		Density	0	0	0.4	0.6	2
		FI	0	0	0.4	0.6	-13.2
		MVI	0	0.6	0.4	0	-11.4
		Z_P.ratio	0.2	0	0	0.8	30.8
	FRic	Community	0.4	0	0.2	0.4	-15.4
		Density	0	0.2	0	0.8	22
		FI	0	0.2	0.2	0.6	4.2
		MVI	0	0.2	0	0.8	-0.8
		Z_P.ratio	0	0	0.2	0.8	21.8
Zooplankton	FDis	Community	0	0	0.6	0.4	10.4
		Density	0.2	0.4	0	0.4	-1
		FI	0	0.2	0.4	0.4	9
		MVI	0	0.4	0.2	0.4	24.6
		Z_P.ratio	0	0	0.4	0.6	0.4
	FEve	Community	0.2	0.2	0.4	0.2	-19
		Density	0.4	0	0.4	0.2	53
		FI	0.4	0	0	0.6	-22
		MVI	0	0	0.2	0.8	6.8
		Z_P.ratio	0.2	0	0.2	0.6	34.4
	FRic	Community	0	0.4	0.4	0.2	-1.4
		Density	0	0	0.4	0.6	21.8
		FI	0	0.6	0.2	0.2	41.8
		MVI	0	0.2	0	0.8	10.6
		Z_P.ratio	0	0.6	0.2	0.2	1.6
